# Supplementary material for: A DNA origami-based aptamer nanoarray for potent and reversible anticoagulation in hemodialysis
Source: Nat Commun. 2021 Jan 13;12:358. doi: 10.1038/s41467-020-20638-7 (PMC7807036; doi:10.1038/s41467-020-20638-7)
Supplement: Supplementary file 1 — Supplementary Information [file 41467_2020_20638_MOESM1_ESM.pdf]

Supplementary Materials for

A DNA origami-based aptamer nanoarray for potent and reversible anticoagulation in hemodialysis

Shuai Zhao,[# 1,2,3] Run Tian,[# 1,2] Jun Wu,[# 4] Shaoli Liu,[1,2] Yuanning Wang,[1] Meng Wen,[4] Yingxu Shang,[1,2] Qing Liu,[1] Yan Li,[1] Ying Guo,[5] Zhaoran Wang,[1,2] Ting Wang,[1] Yujing Zhao,[4] Huiru Zhao,[4] Hui Cao,[4] Yu Su,[4] Jiashu Sun,[1,2] Qiao Jiang*[1,2] and Baoquan Ding*[1,2,6]

[1] CAS Key Laboratory of Nanosystem and Hierarchical Fabrication, CAS Center for Excellence in Nanoscience, National Center for Nanoscience and Technology, Beijing 100190, China

[2] University of Chinese Academy of Sciences, Beijing 100049, China

[3] Sino-Danish College, Sino-Danish Center for Education and Research, University of Chinese Academy of Sciences, Beijing 100049, China

[4] Department of laboratory medicine, Peking University Fourth School of Clinical Medicine, Beijing Jishuitan Hospital, Beijing 100035, China

[5] National & Local Joint Engineering Research Center of Biodiagnosis and Biotherapy, The Second Affiliated Hospital of Xi'an Jiaotong University, Xi'an 710004, China

[6] School of Materials Science and Engineering, Zhengzhou University, Zhengzhou 450001, China

[#]These authors contributed equally to this work.

*Correspondence to: jiangq@nanoctr.cn, dingbq@nanoctr.cn

1. Supplementary Figures (S1 - S38)

1.1 Design and characterization of DNA origami-aptamer nanostructures (S1 - S18)

1.2 Supplementary characterization of thrombin inhibition by DNA origami-aptamer nanostructures in the reaction mixtures (S19 - S24)

1.3 Additional anticoagulation of Aptarray in blood-related samples (S25 - S33)

1.4 Safety assessment of the Aptarray (S34 - S38)

2. Supplementary Tables (T1 - T2)

1. Supplementary Figures

1.1 Design and characterization of DNA origami-aptamer nanostructures

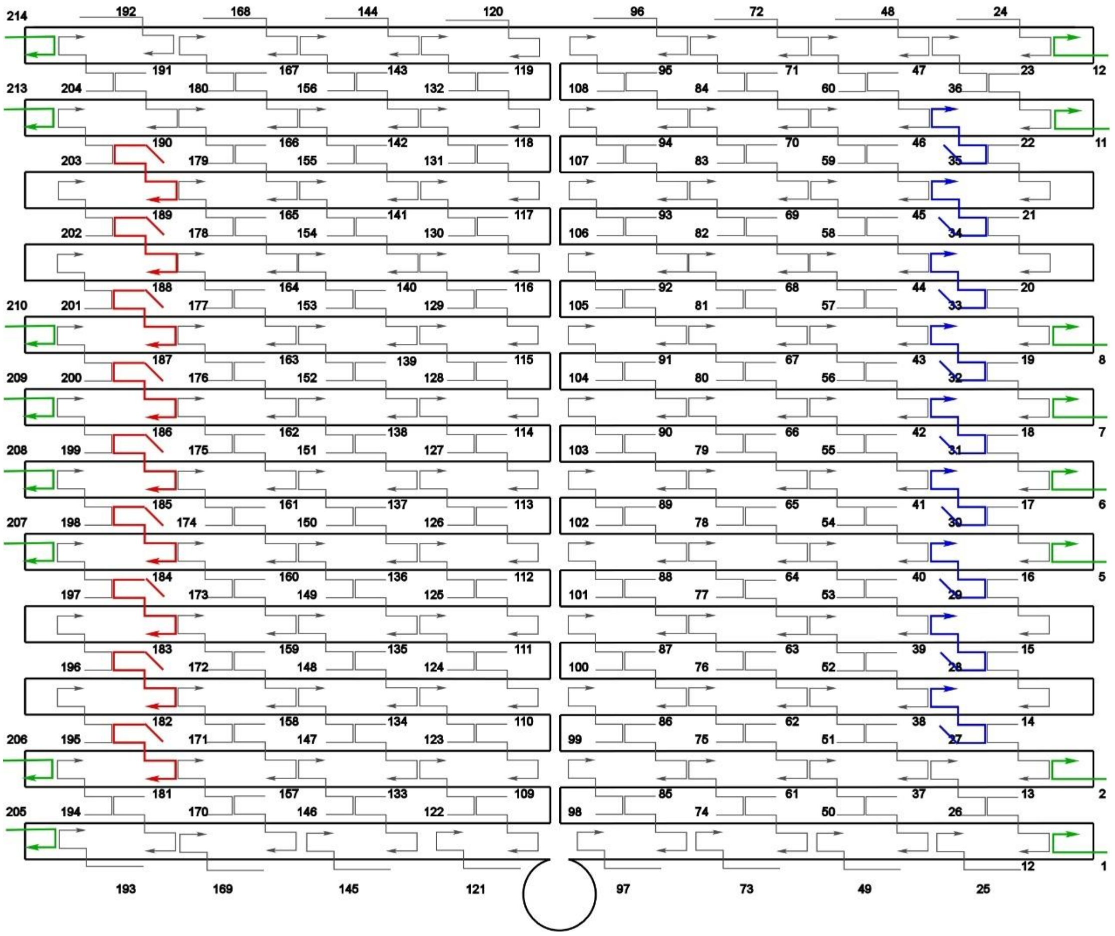

Supplementary Figure 1. DNA origami design (I) with functional strands for thrombin-binding aptamer loading. The design of the rectangular DNA origami structure with M13 phage single-stranded DNA (black) and staple strands (gray) is shown. Nine loading strands in blue (27, 28, 29, 30, 31, 32, 33, 34 and 35) or red (182, 183, 184, 185, 186, 187, 188, 189 and 190) are extended at their 5'-end with different ssDNA sequences to capture TBA15 or HD22, respectively. The two types of binding sites were with a distance of ~ 68 nm. To avoid a stacking effect during assembly, staple strands in green along the two narrow sides (1, 2, 5, 6, 7, 8, 11, 12, 205, 206, 207, 208, 209, 210, 213 and 214) are extended with TTTTT at their 5'-ends.

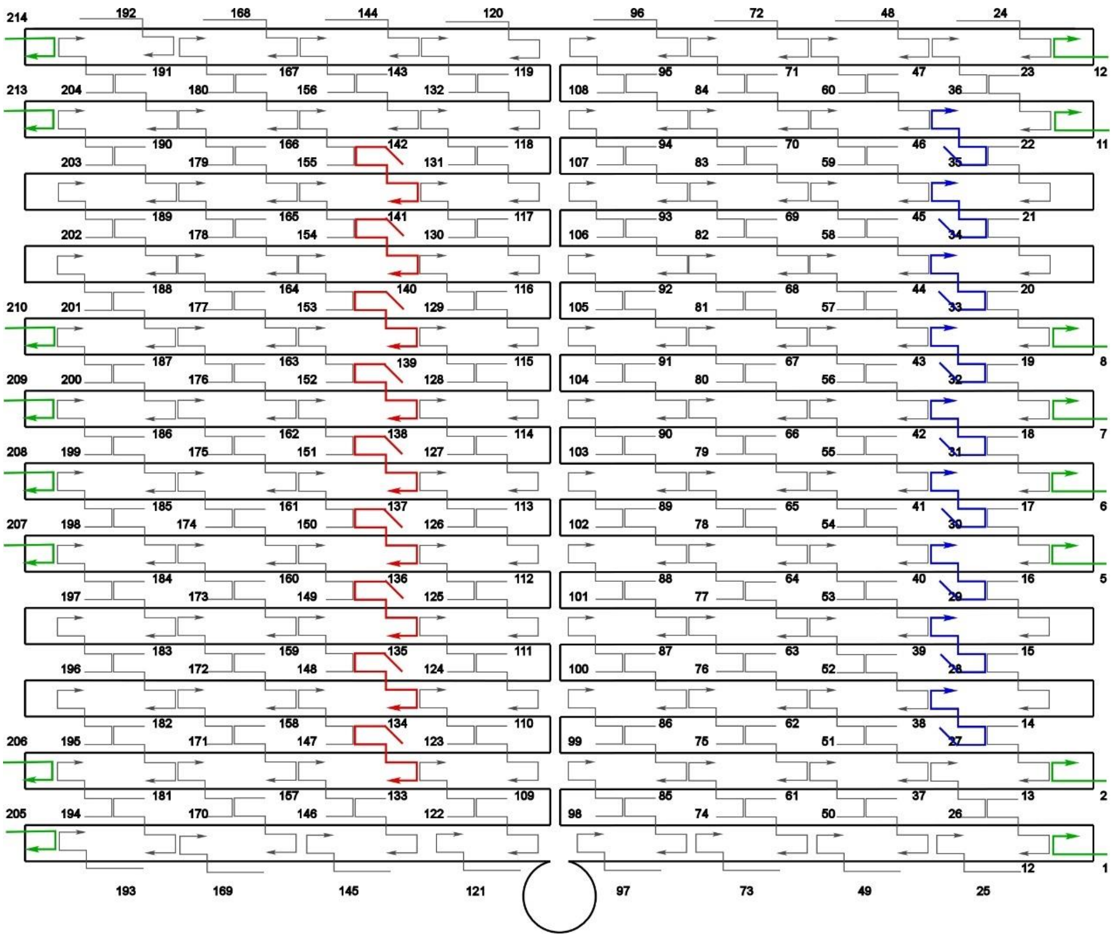

Supplementary Figure 2. DNA origami design (II) with functional strands for aptamer loading. Nine loading strands in blue (27, 28, 29, 30, 31, 32, 33, 34 and 35) or red (134, 135, 136, 137, 138, 139, 140, 141 and 142) are extended at their 5'-end with ssDNA sequences to capture TBA15 or HD22, respectively. The two types of binding sites were with a distance of ~ 46 nm.

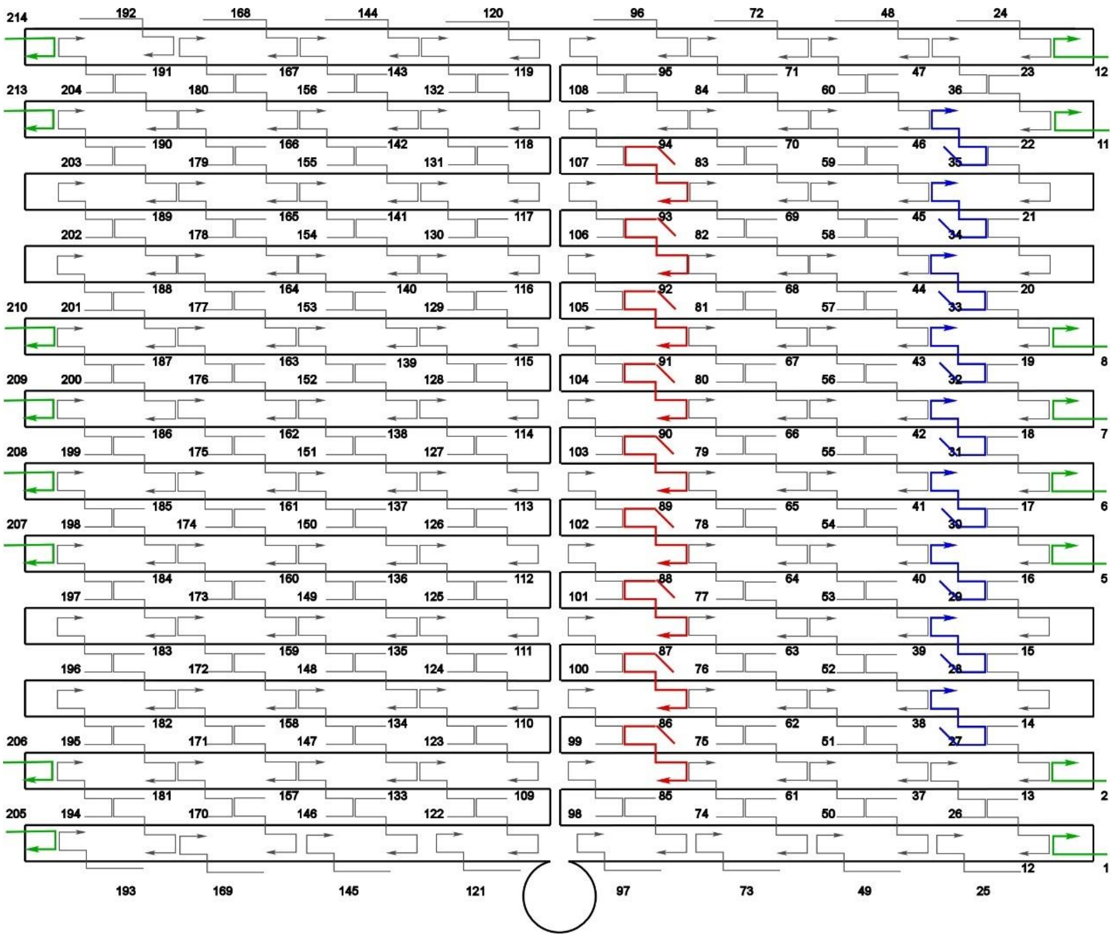

Supplementary Figure 3. DNA origami design (III) with functional strands for aptamer loading. Nine loading strands in blue (27, 28, 29, 30, 31, 32, 33, 34 and 35) or red (86, 87, 88, 89, 90, 91, 92, 93 and 94) are extended at their 5'-end with ssDNA sequences to capture TBA15 or HD22, respectively. The two types of binding sites were with a distance of ~ 24 nm.

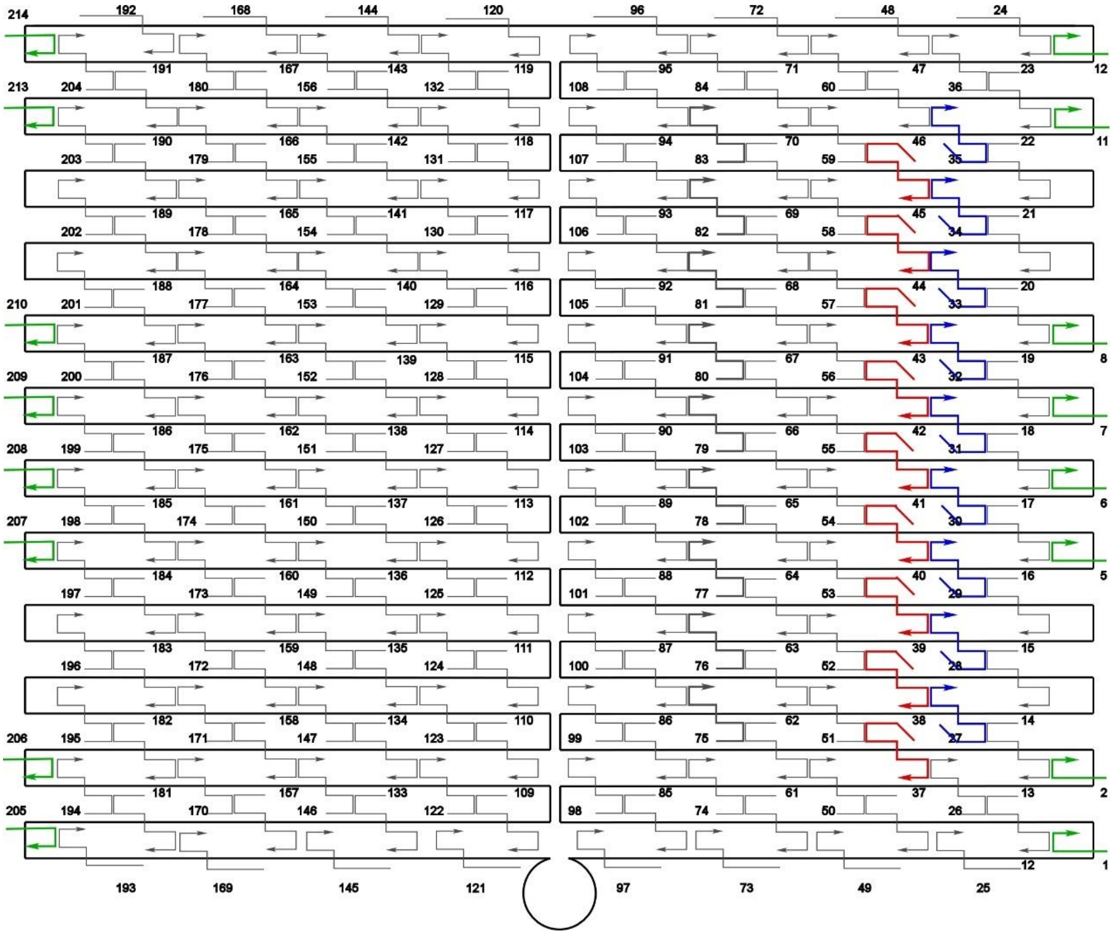

Supplementary Figure 4. DNA origami design (IV) with functional strands for aptamer loading. Nine loading strands in blue (27, 28, 29, 30, 31, 32, 33, 34 and 35) or red (38, 39, 40, 41, 42, 43, 44, 45 and 46) are extended at their 5'-end with ssDNA sequences to capture TBA15 or HD22, respectively. The two types of binding sites were with a distance of ~ 5.4 nm.

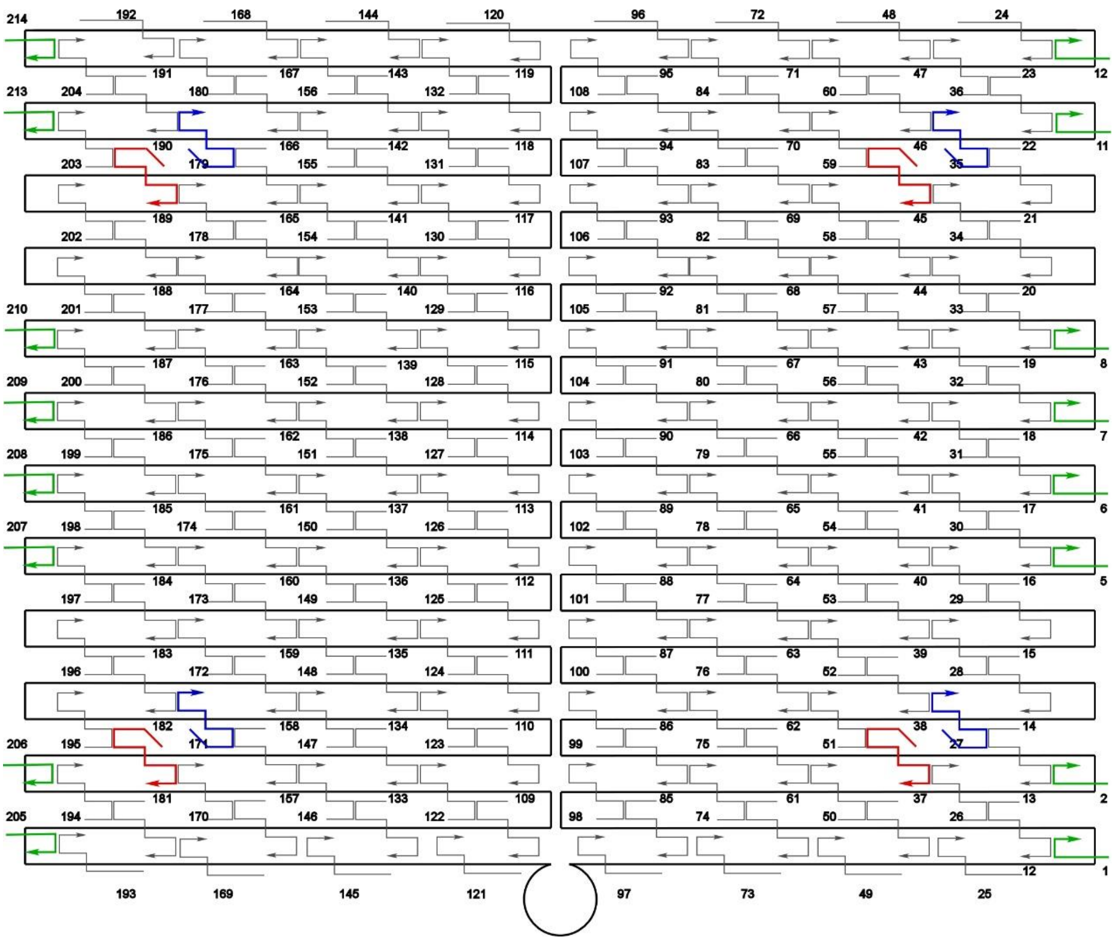

Supplementary Figure 5. DNA origami design (V) with functional strands for aptamer loading. Four loading strands in blue (27, 35, 171 and 179) or red (38, 46, 182 and 190) are extended at their 5’-end with different ssDNA sequences to capture TBA15 or HD22, respectively. The two types of binding sites were with a distance of ~ 5.4 nm.

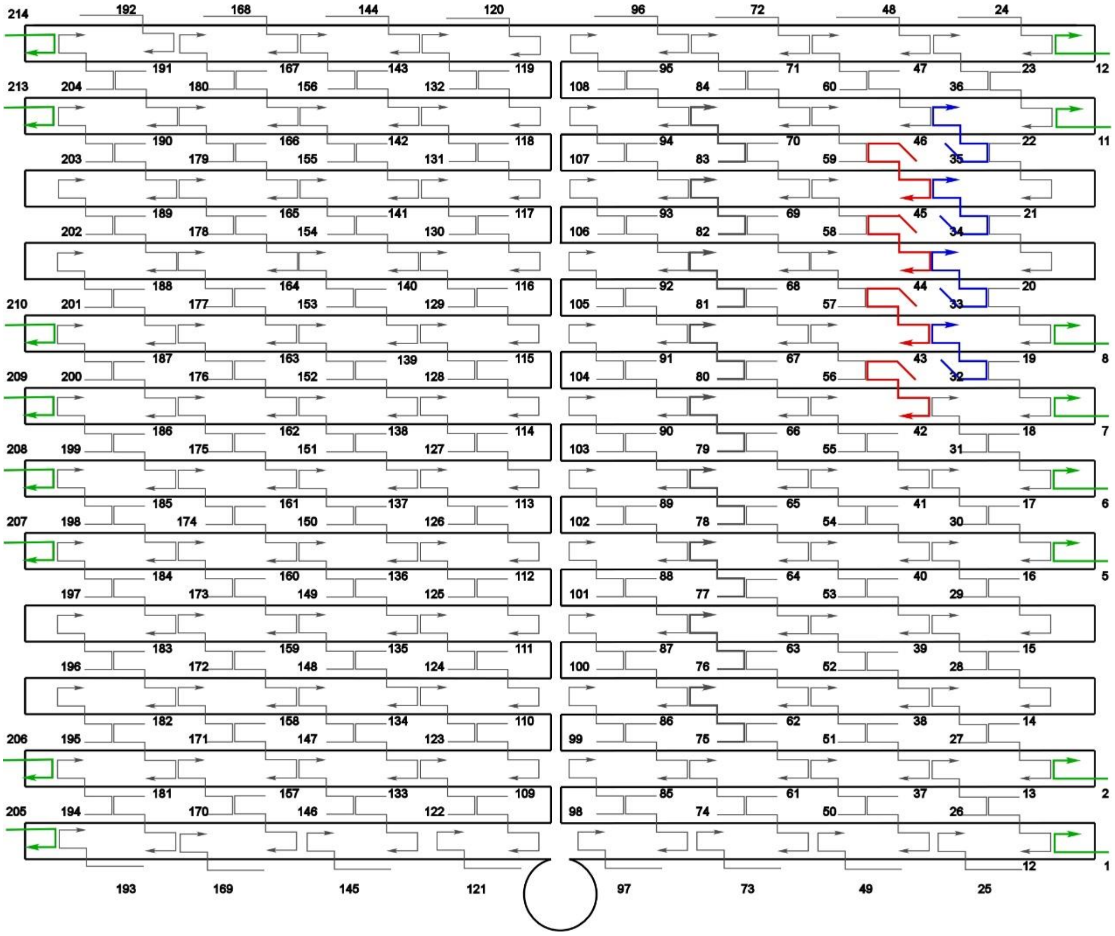

Supplementary Figure 6. DNA origami design (VI) with functional strands for aptamer loading. Four loading strands in blue (32, 33, 34 and 35) or red (43, 44, 45 and 46) are extended at their 5'-end with different ssDNA sequences to capture TBA15 or HD22, respectively. The two types of binding sites were with a distance of ~ 5.4 nm.

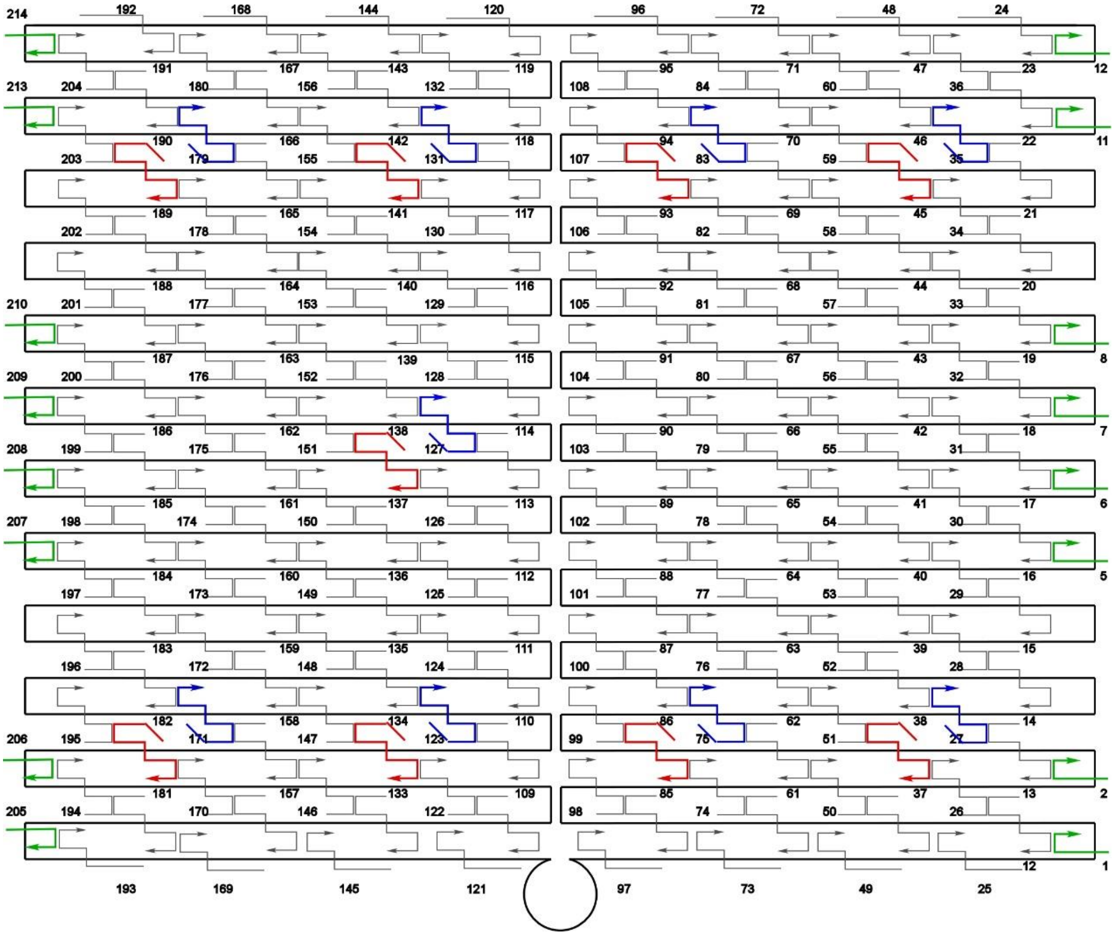

Supplementary Figure 7. DNA origami design (VII) with functional strands for aptamer loading. Nine loading strands in blue (27, 35, 75, 83, 123, 127, 131, 171 and 179) or red (38, 46, 86, 94, 134, 138, 142, 182 and 190) are extended at their 5’-end with different ssDNA sequences to capture TBA15 or HD22, respectively. The two types of binding sites were with a distance of ~ 5.4 nm.

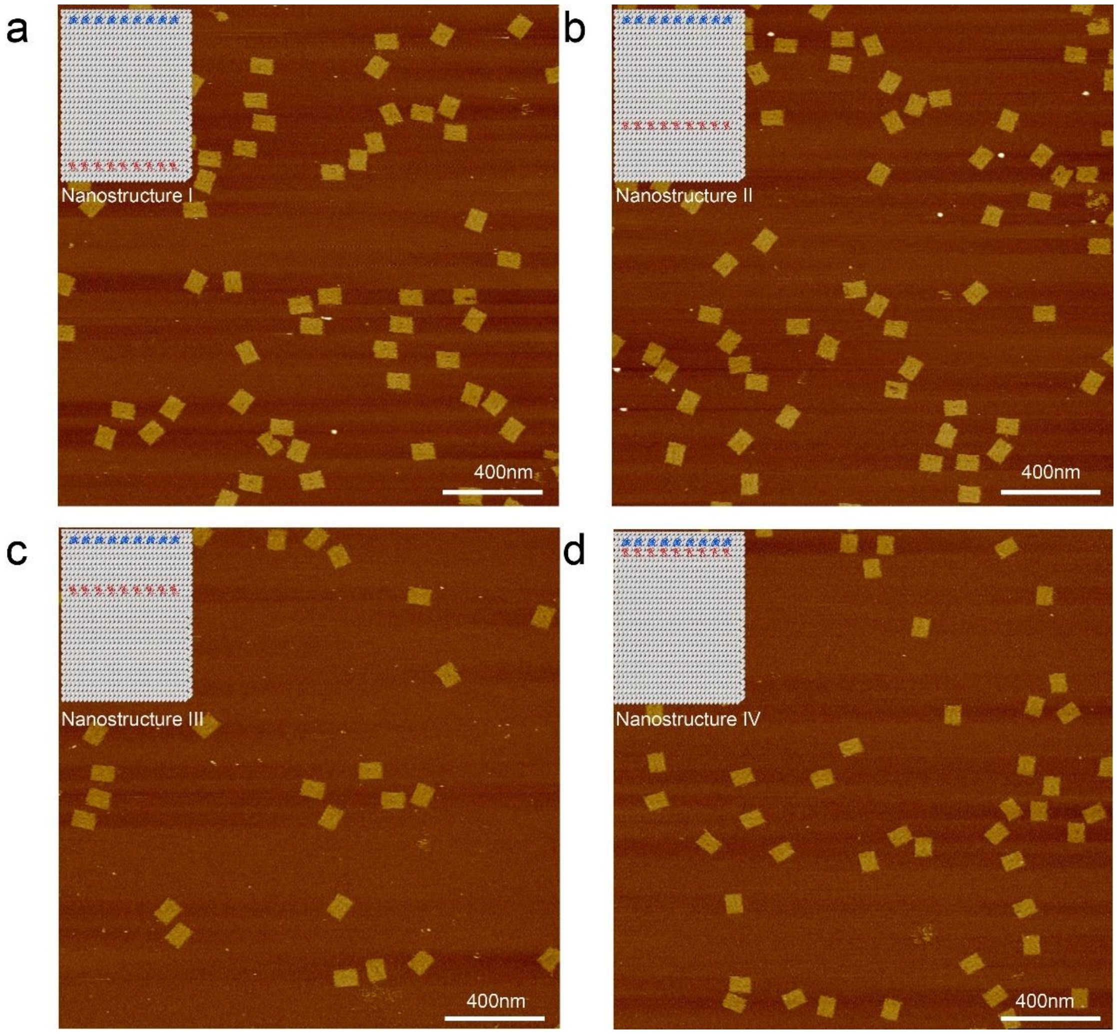

Supplementary Figure 8. AFM images of aptamer-extending DNA origami nanostructures. (a) Nanostructure I, (b) Nanostructure II, (c) Nanostructure III and (d) Nanostructure IV. Scale bars, 400 nm. The AFM images are representative of three independent experiments.

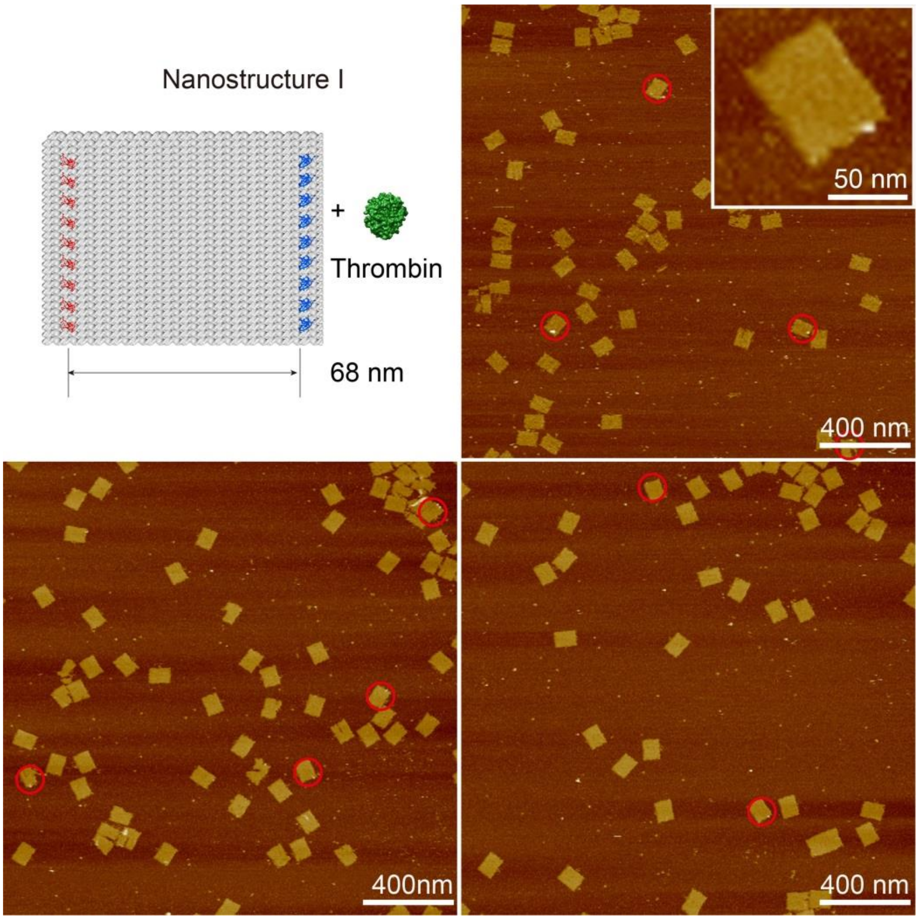

Supplementary Figure 9. Additional AFM images of aptamer-extending DNA origami nanostructure I (the distance of two types of aptamers is ~ 68 nm) after incubation with thrombin molecules. The red circles indicate the thrombin binding on the DNA origami nanostructures. Scale bars, 400 nm. The AFM images are representative of three independent experiments.

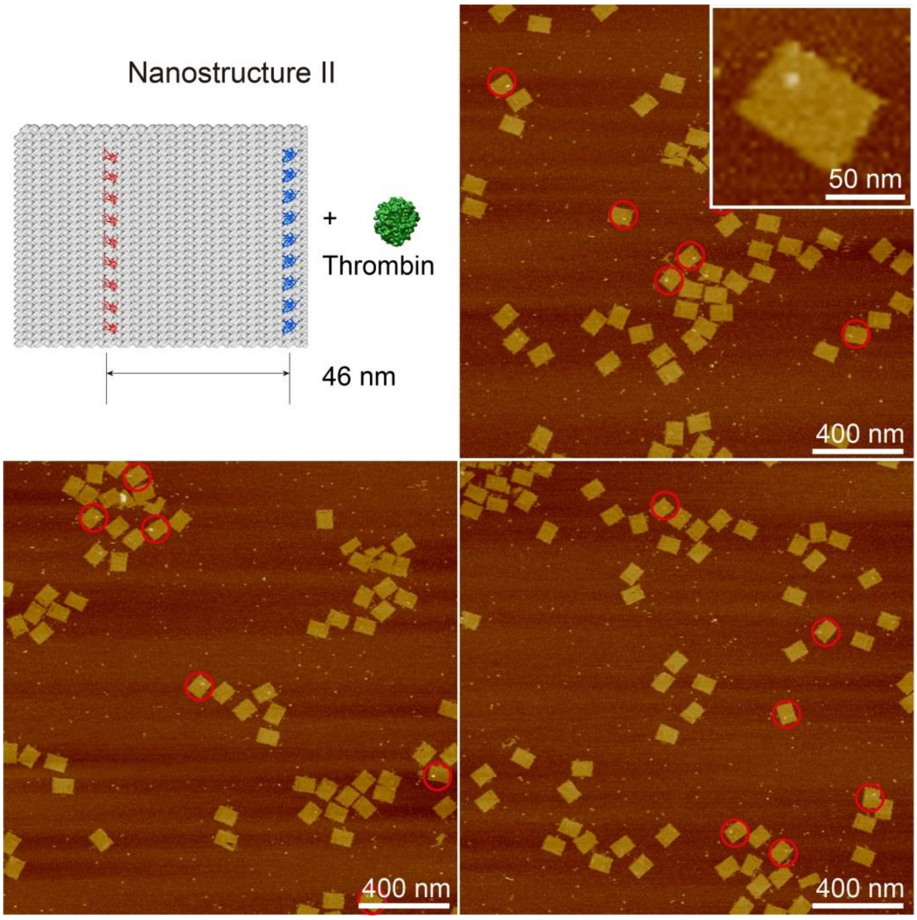

Supplementary Figure 10. Additional AFM images of aptamer-extending DNA origami nanostructure II (the distance of two types of aptamers is ~ 46 nm) after incubation with thrombin molecules. The red circles indicate the thrombin binding on the DNA origami nanostructures. Scale bars, 400 nm. The AFM images are representative of three independent experiments.

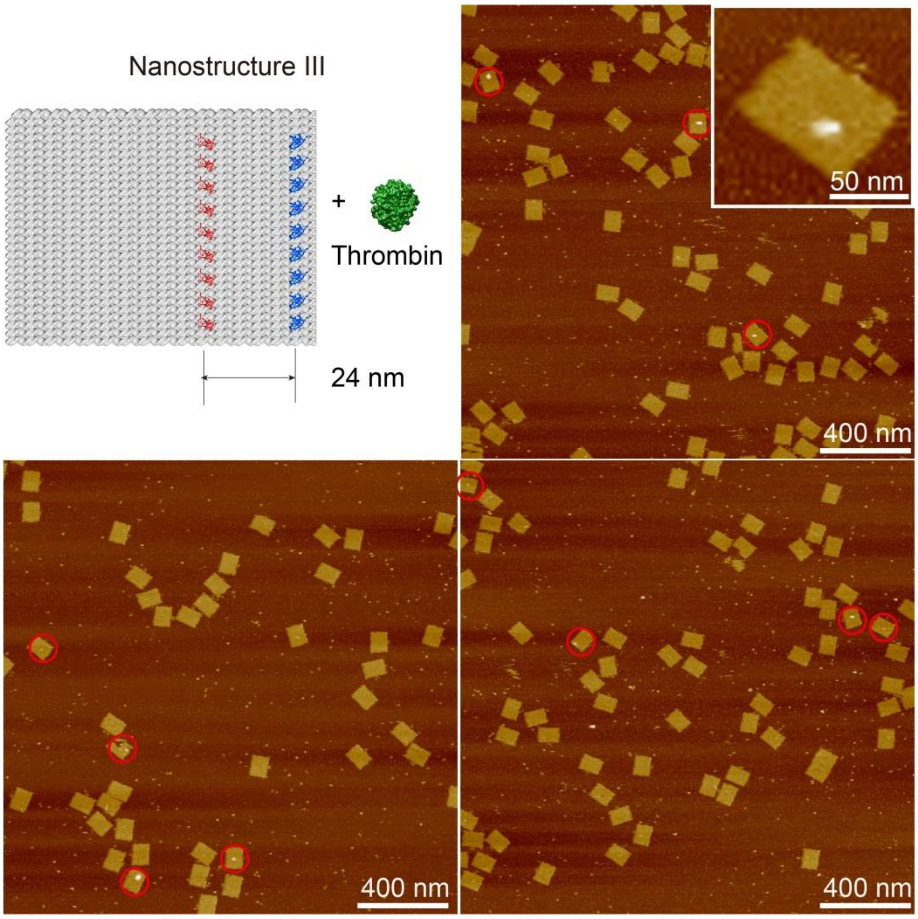

Supplementary Figure 11. Additional AFM images of aptamer-extending DNA origami nanostructure III (the distance of two types of aptamers is ~ 24 nm) after incubation with thrombin molecules. The red circles indicate the thrombin binding on the DNA origami nanostructures. Scale bars, 400 nm. The AFM images are representative of three independent experiments.

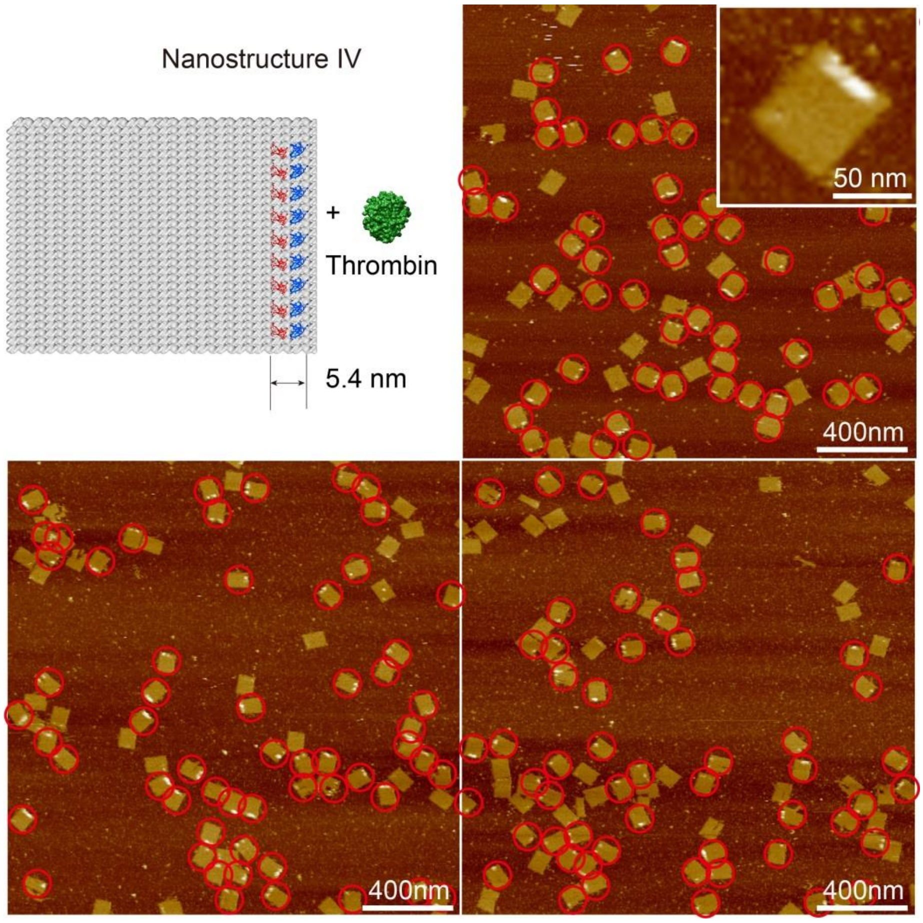

Supplementary Figure 12. Additional AFM images of aptamer-extending DNA origami nanostructure IV (the distance of two types of aptamers is ~ 5.4 nm) after incubation with thrombin molecules. The red circles indicate the thrombin binding on the DNA origami nanostructures. Scale bars, 400 nm. The AFM images are representative of three independent experiments.

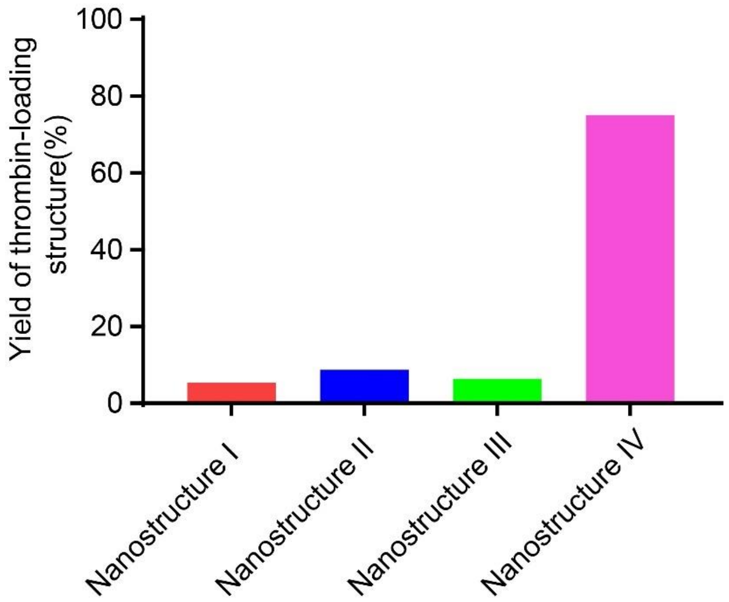

Supplementary Figure 13. The yield of thrombin binding on the DNA origami-aptamer nanostructure I-IV from the AFM images in Supplementary Figure 9-12. The efficiency of thrombin loading was calculated from AFM images (Supplementary Figure 9-12, containing > 150 origami structures) by dividing the number of thrombin-binding structures (highlighted by the red circle) by the total number of origami assemblies counted. Approximately 76% of the DNA nanostructure IV contained thrombin molecules on the surface.

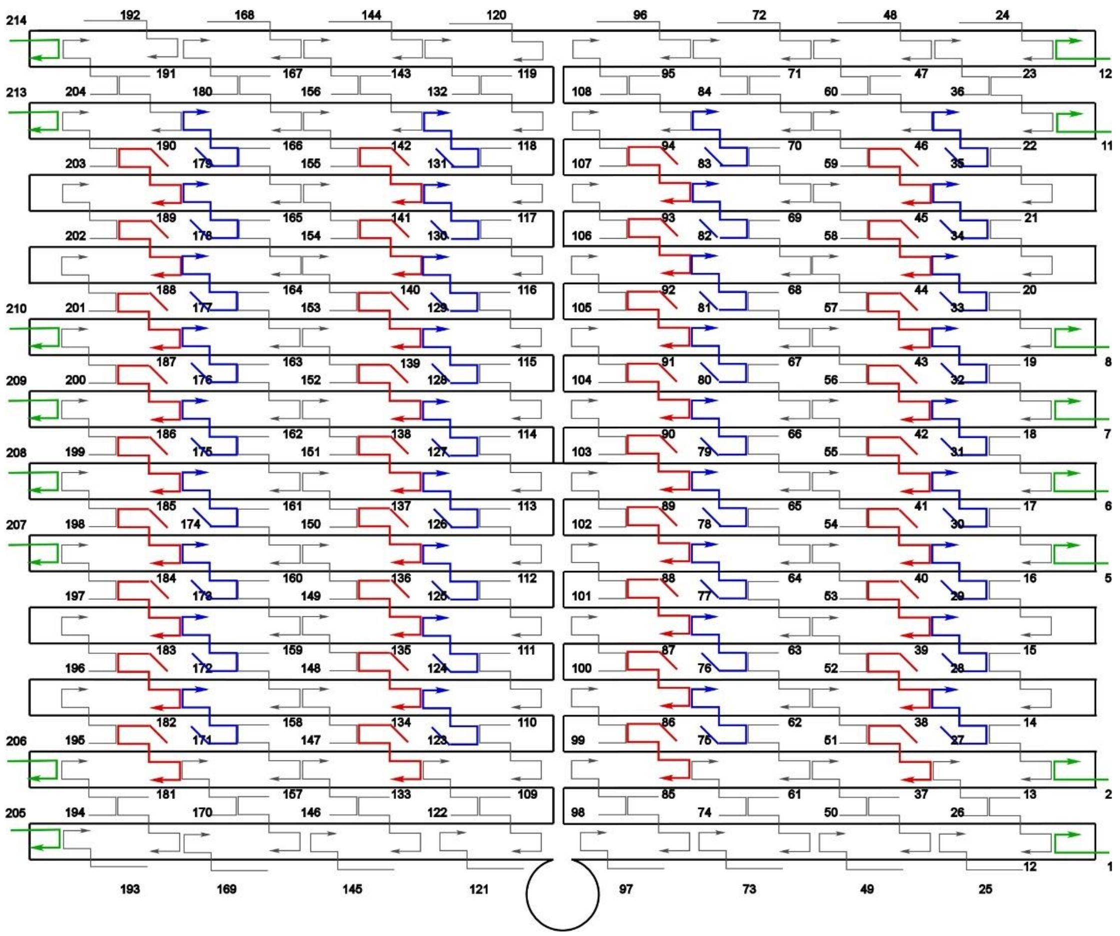

Supplementary Figure 14. DNA origami design with functional strands arranged in four-line array for aptamer loading. Thirty-six loading strands in blue (27-35, 76-84, 123-131 and 171-179) or red (38-46, 86-94, 134-142 and 182-190) are extended at their 5'-end with different ssDNA sequences to capture TBA15 or HD22, respectively. The two types of binding sites were with distance of ~ 5.4 nm. To avoid a stacking effect during assembly, staple strands in green along the two narrow sides (1, 2, 5, 6, 7, 8, 11, 12, 205, 206, 207, 208, 209, 210, 213 and 214) are extended with TTTTT at their 5'-ends.

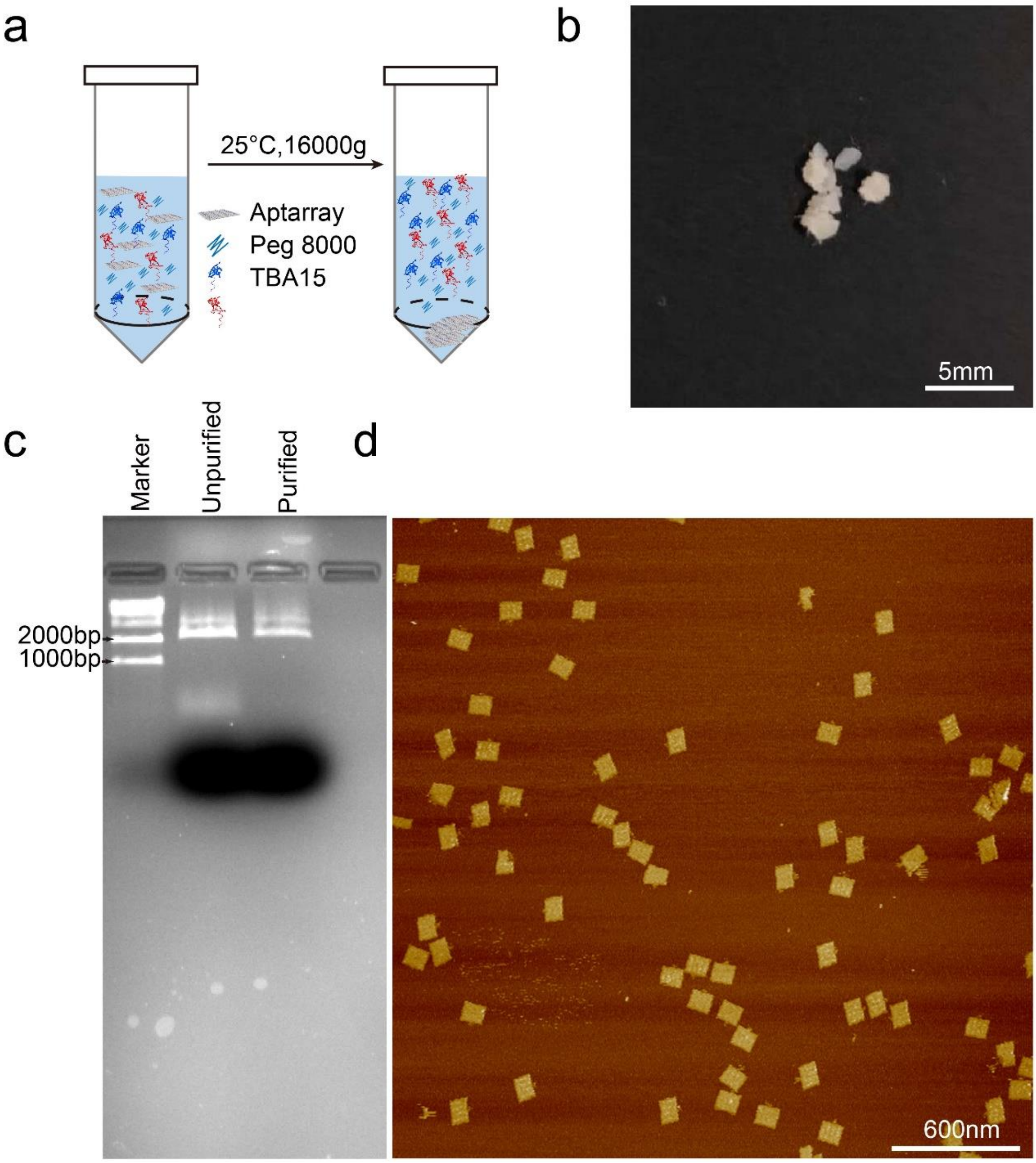

Supplementary Figure 15. PEG purification of DNA origami-aptamer nanoarray (containing 36 TBA-15 and 36 HD22, Aptarray). (a) Schematic illustration of the PEG purification procedure for Aptarray. (b) Approximately 4.3 mg of solid materials containing Aptarray nanostructures. Scale bar, 5 mm. (c) Agarose gel image of unpurified (center) Aptarray and PEG-purified, re-dissolved (right) Aptarray. (d) AFM micrograph of dried and re-dissolved Aptarray. Scale bar, 600 nm. The sample graph (b), gel results (c) and AFM image (d) are representative of three independent experiments.

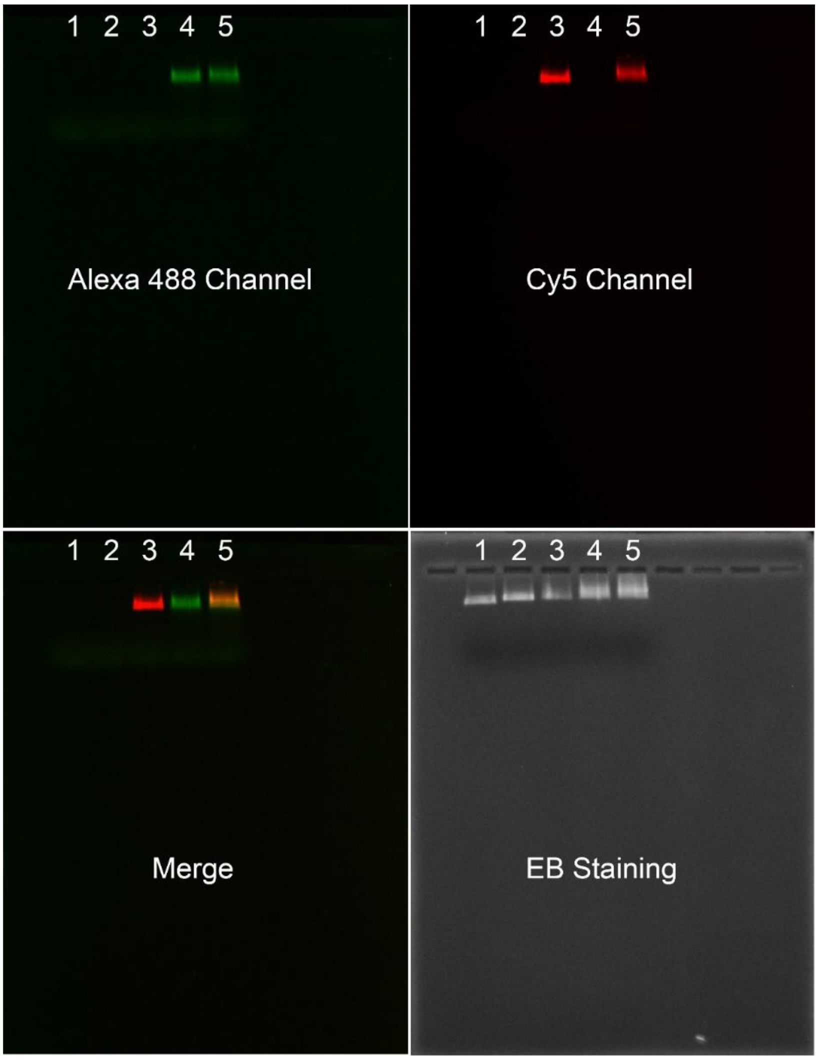

Supplementary Figure 16. Agarose gel images of DNA origami-aptamer nanoarray. Lane 1, M13 genome DNA. Lane 2, bare rectangular DNA origami. Lane 3, Cy5-TBA15 loaded DNA origami. Lane 4, Alexa 488-HD22 loaded origami. Lane 5, Cy5-TBA15 and Alexa 488-HD22 co-loaded origami, Aptarray. The DNA origami co-migrated with Cy5-TBA15 and Alexa 488-HD22, indicating the binding of two types of aptamers to the DNA origami nanostructures. The gel images are representative of three independent experiments.

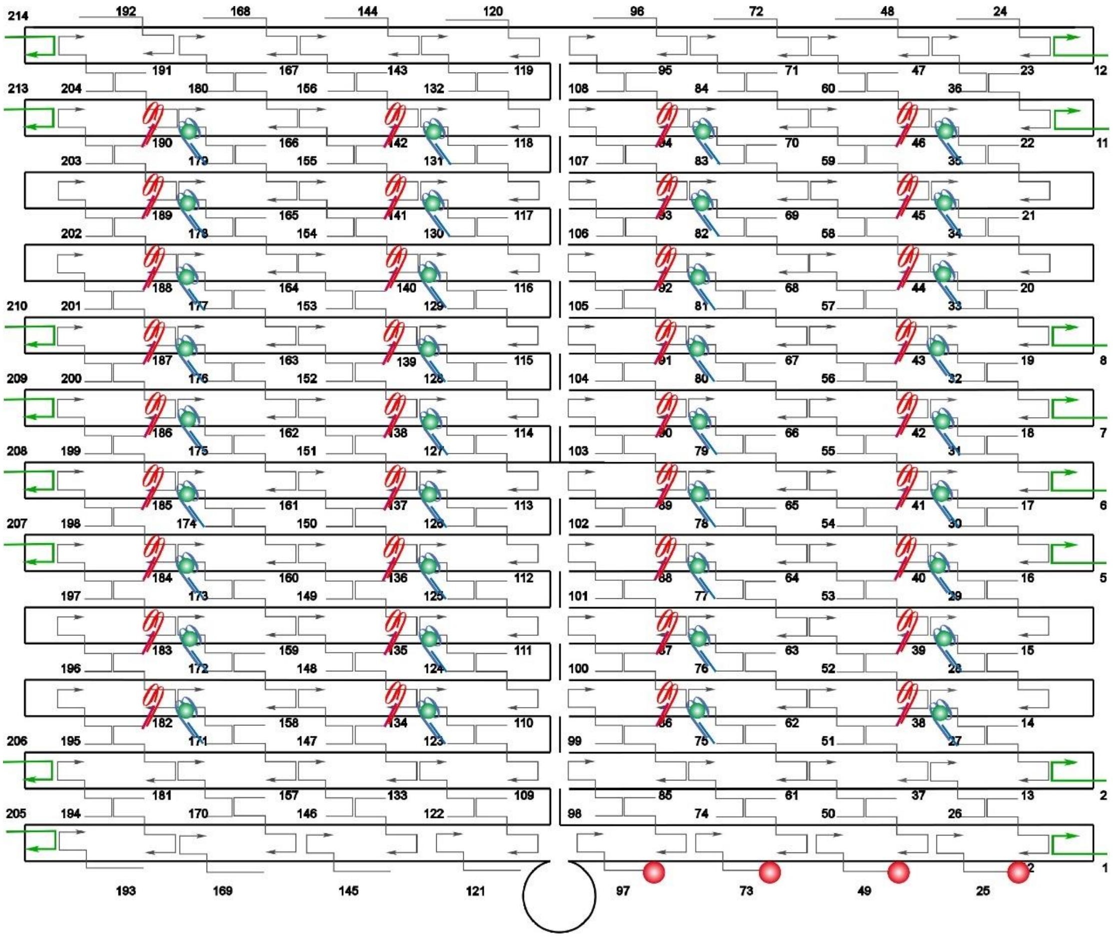

Supplementary Figure 17. The loading efficiency of aptamers on DNA origami. Four Cy5-modified staple strands (25, 49, 73 and 97) were fabricated to rectangular DNA origami as a reference to calculate the concentration of DNA origami. The Cy5-origami was quantified by measuring the absorbance at 650 nm (A650) using an extinction coefficient of 2.5×10^5 $M^{-1}cm^{-1}$. Two types of aptamers (one type of them was labeled with Alexa488) were hybridized with captures extending DNA origami. The Alexa488-aptamer was estimated by measuring the absorbance at 488 nm (A488) using an extinction coefficient of 6.5×10^4 $M^{-1}cm^{-1}$.

Ratio (TBA15/origami) = $C_{Alexa488\text{-}TBA15}/C_{Cy5\text{-}origami} = 33.5 \pm 0.4$

Ratio (HD22/origami) = $C_{Alexa488\text{-}HD22}/C_{Cy5\text{-}origami} = 32.4 \pm 1.1$

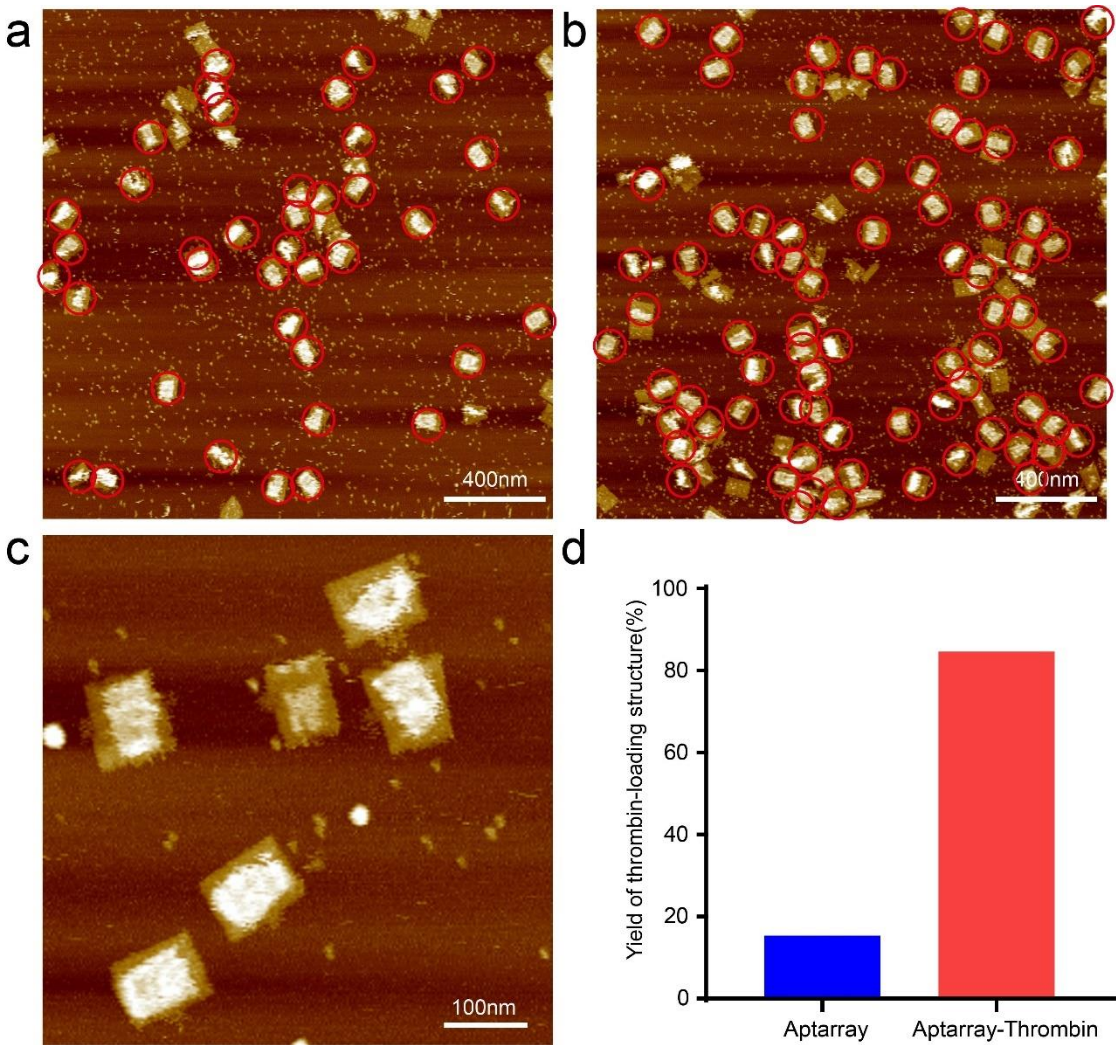

Supplementary Figure 18. Additional AFM micrographs of Aptarray after thrombin incubation. (a-c) The majority of Aptarray structures were covered by the large area of bright spots on the surface, representing the captured enzyme molecules. The little spots scattered in the images are the redundant thrombin molecules. Scale bars in (a-b), 400 nm; in (c) 100 nm. (d) The efficiency of thrombin loading was calculated from AFM images (a-b, containing > 150 Aptarrays) by dividing the number of thrombin-binding structures (highlighted by the red circle) by the total number of Aptarrays counted. Approximately 85% of the Aptarray was covered by thrombin molecules on the surface. The AFM images are representative of three independent experiments.

1.2 Supplementary characterization of thrombin inhibition by DNA origami-aptamer nanostructures in the reaction mixtures

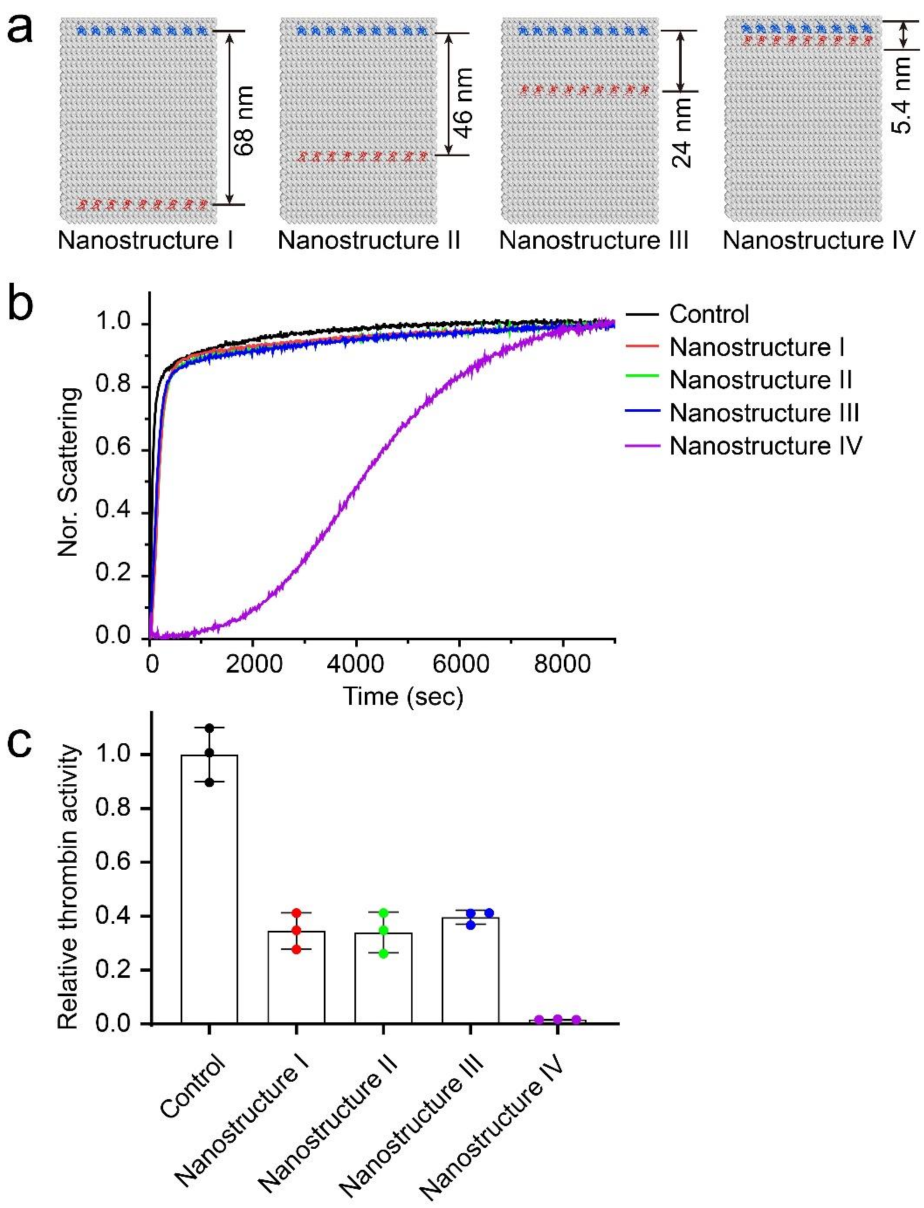

Supplementary Figure 19. Distance-dependent thrombin inhibition. (a) Schematic drawing of four different DNA nanostructures I-IV (with two rows of nine of each aptamer arranged ~ 68 nm, 46 nm, 24 nm or 5.4 nm apart, respectively). (b) Light scatting spectra (λsc = 650 nm) of fibrinogen solution with thrombin only (Control) or thrombin inhibited by aptamers extending origami nanostructures I-IV. (c) Relative thrombin activities of different treatments were estimated by the catalytic rate of thrombin (V_{Cat}) obtained from the light scattering intensities (b). The catalytic rate of thrombin (V_{Cat}) after the indicated treatments was calculated as $V_{Cat} = C_{Fibrinogen} / (t_{1/2} \times C_{Thrombin})$ obtained from the panel (b). The concentration of fibrinogen is 1 mg/ml, and the concentration of thrombin is 12 nM. Data represent the mean ± s.d. of three independent experiments.

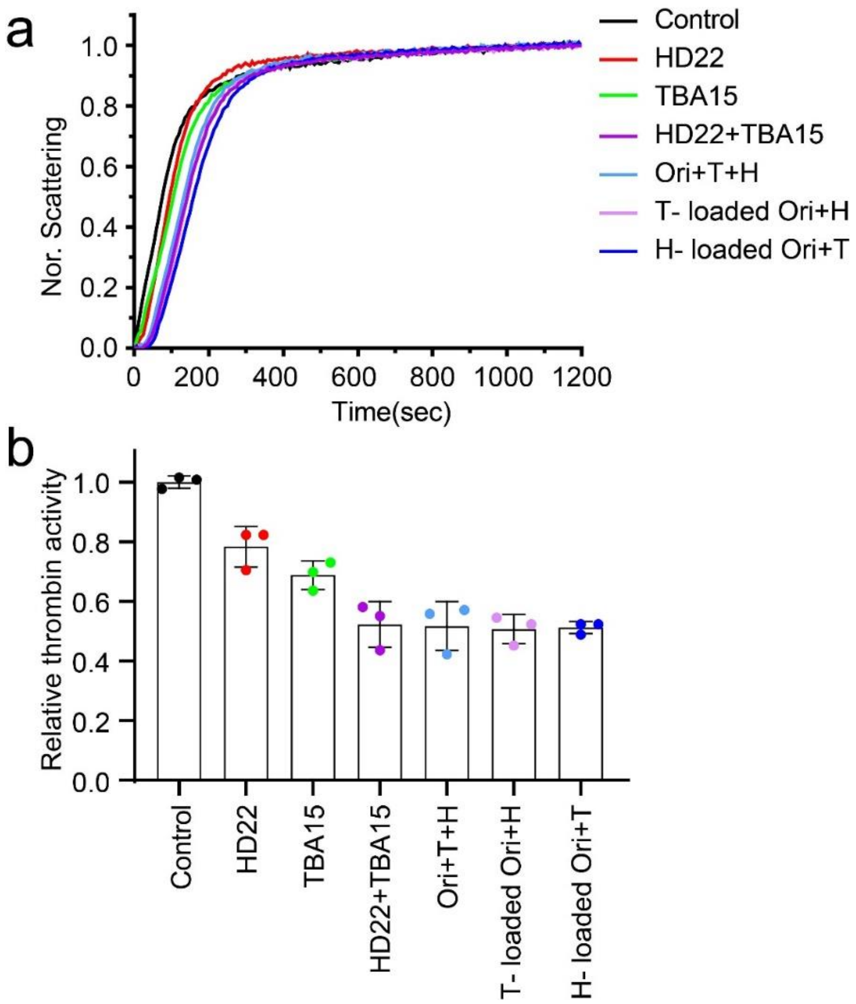

Supplementary Figure 20. Thrombin inhibition by different control groups. (a) Light scatting spectra of fibrinogen solution with thrombin only (Control) or thrombin inhibited by free aptamers (HD22, TBA15), the mixtures of two types of aptamers (HD22 + TBA15), the mixtures of the aptamers and rectangular origami templates (Ori + T + H), TBA15-loaded origami plus HD22 (T-loaded Ori + H), and HD22-loaded origami plus TBA15 (H-loaded Ori + T) at equivalent aptamer amounts. (b) Relative thrombin activities after different treatments were calculated by the results of (a). Data represent the mean ± s.d. of three independent experiments.

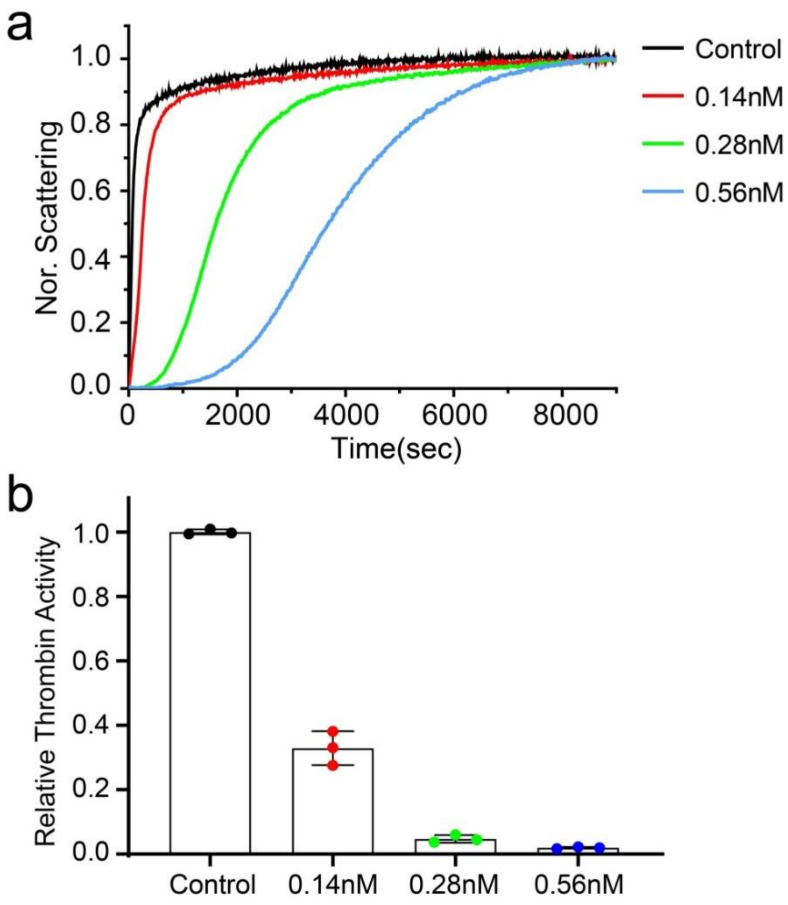

Supplementary Figure 21. Dose-dependent thrombin inhibition. (a) Light scatting spectra of fibrinogen solution with thrombin only (Control) or thrombin inhibited by Aptarray with different concentration (~ 0.14 nM Aptarray, containing 5 nM TBA15, 5 nM HD22; ~ 0.28 nM Aptarray, containing 10 nM TBA15, 10 nM HD22; ~ 0.56 nM Aptarray, containing 20 nM TBA15, 20 nM HD22). (b) Relative thrombin activities after different treatments were calculated by the results of (a). Data represent the mean ± s.d. of three independent experiments.

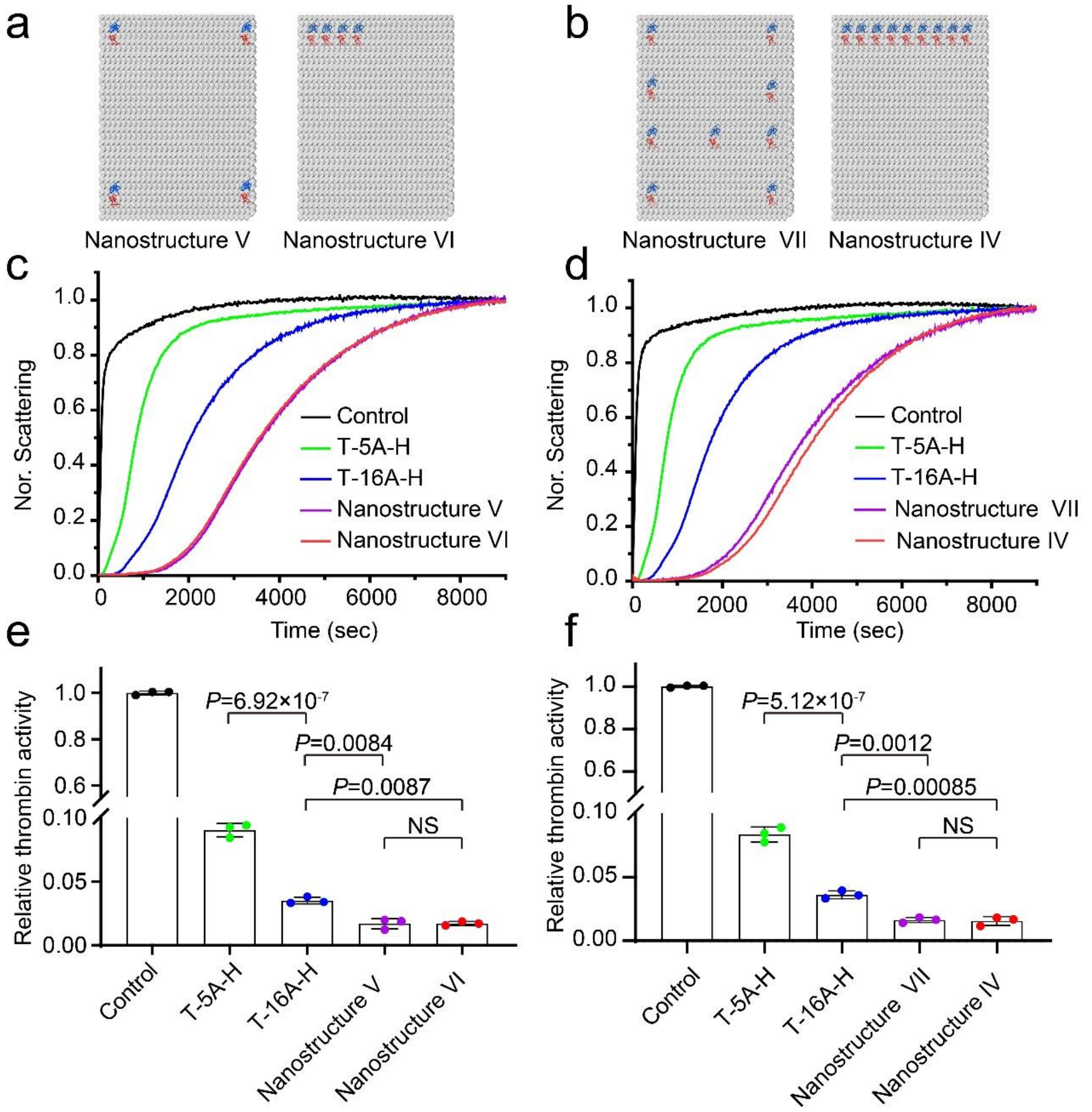

Supplementary Figure S22. Thrombin inhibition of the nanostructures with different bi-aptamer densities or numbers. (a-b) Schematic representation of structures with different bi-aptamer patterns. (c-d) Light scatting spectra (λsc = 650 nm) of a fibrinogen solution with thrombin only (Control) or thrombin inhibited by aptamers tethered with 5-mer (T-5A-H, 20 nM), 16-mer polyA linkers (T-16A-H, 20 nM), Nanostructure V, VI (c, ~ 5 nM origami, 20 nM TBA15, 20 nM HD22), VII or IV (d, ~2.2 nM origami, 20 nM TBA15, 20 nM HD22). (e-f) Relative thrombin activities of different treatments were estimated by the catalytic rate of thrombin with indicated groups. The data (e, f) represent the mean ± s.d. of three independent experiments. Statistical significance (e, f) was calculated by one-way ANOVA with the Tukey post hoc test. NS, $P > 0.05$.

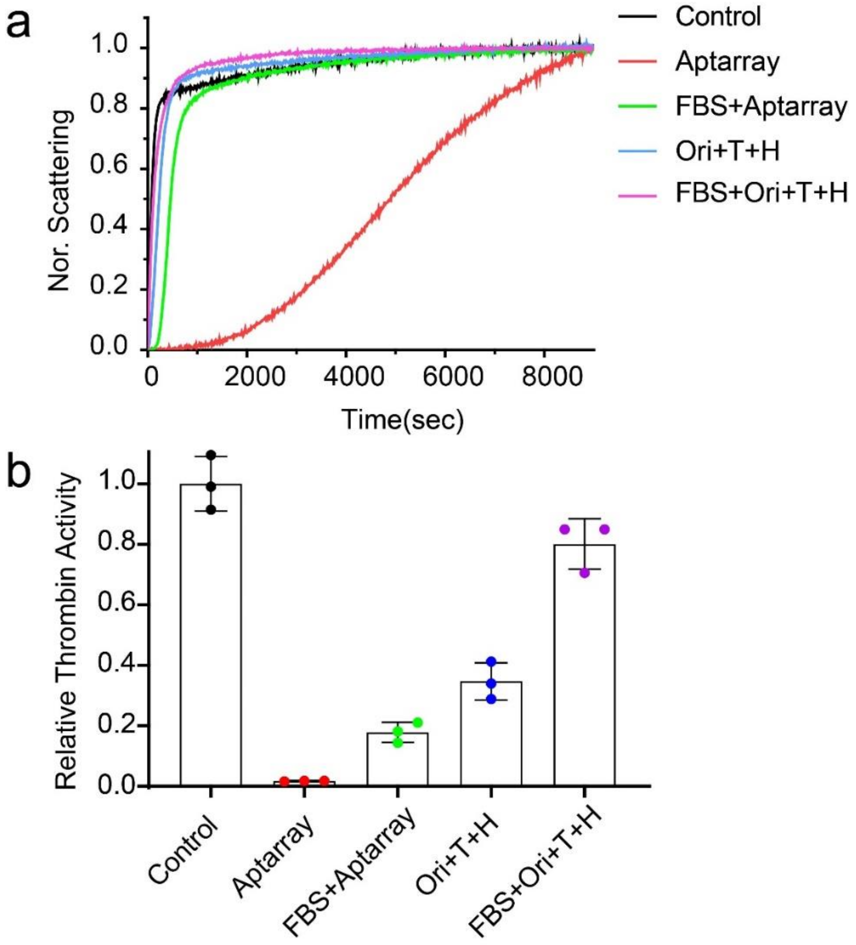

Supplementary Figure 23. Clotting reaction before or after fetal bovine serum (FBS) incubation. (a) Light scatting spectra of fibrinogen solution with thrombin only (Control), thrombin inhibited by the mixture of DNA origami and two types of aptamers (Ori + T + H), by FBS-pre-treated mixture (FBS + Ori + T + H), by Aptarray, and by FBS-incubated Aptarray (FBS + Aptarray). (b) Relative thrombin activities after different treatments were calculated by the results of (a). Data represent the mean ± s.d. of three independent experiments.

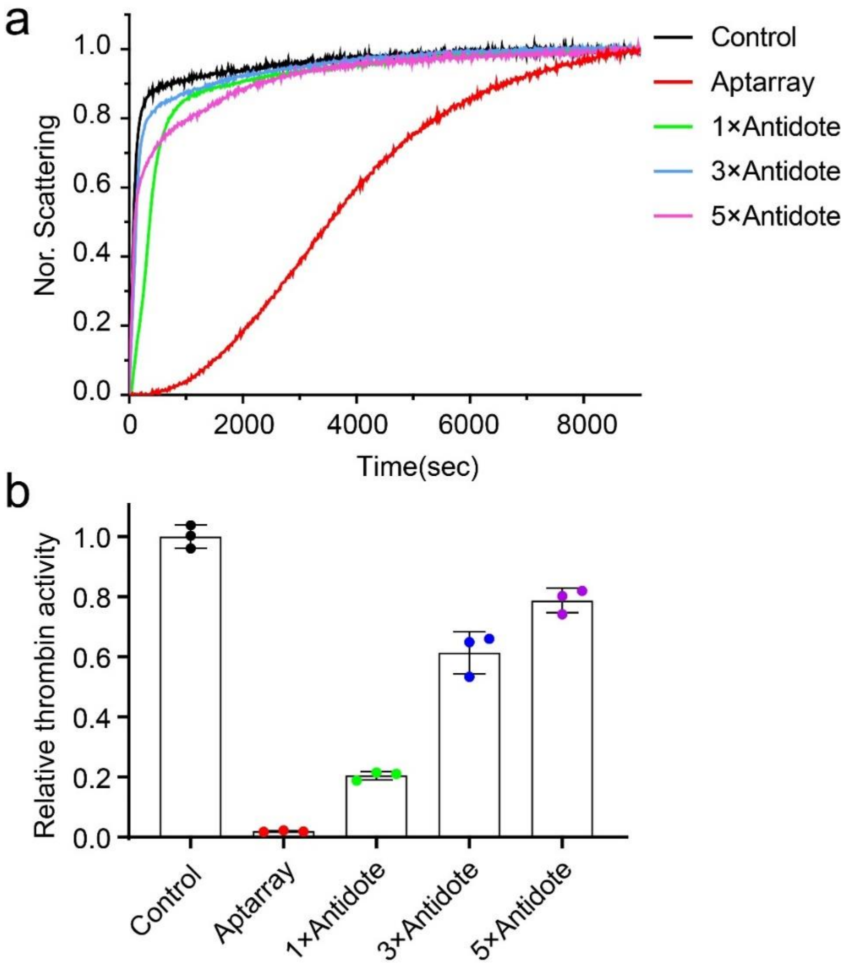

Supplementary Figure 24. Inhibitor neutralization of clotting reaction. (a) Light scatting spectra of fibrinogen solution with thrombin only (Control), thrombin inhibited by Aptarray or by antidote strands mixtures (cTBA15 + cHD22)-treated Aptarray (1-fold antidote, 3-fold antidote, 5-fold antidote). (b) Relative thrombin activities after different treatments were calculated by the results of (a). Data represent the mean ± s.d. of three independent experiments.

1.3 Additional anticoagulation of Aptarray in blood-related samples

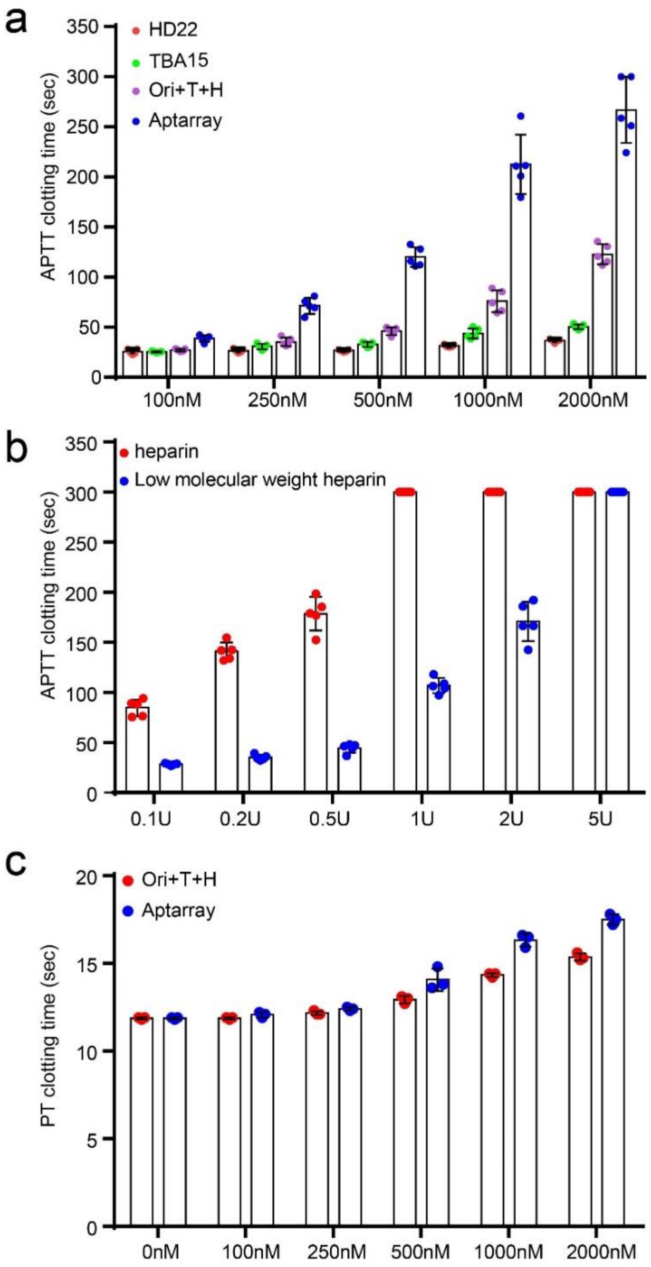

Supplementary Figure 25. Activated partial thromboplastin time (APTT) and prothrombin time (PT) of human plasma clotting measurements. (a) APTT of human plasma clotting in presence of HD22, TBA15, the mixture of origami and aptamers (Ori + T + H) or Aptarray with different concentration (~ 2.8 nM origami, 100 nM TBA15, 100 nM HD22; ~ 7 nM origami, 250 nM TBA15, 250 nM HD22; ~ 14 nM origami, 500 nM TBA15, 500 nM HD22; ~ 28 nM origami, 1000 nM TBA15, 1000 nM HD22; ~ 56 nM origami, 2000 nM TBA15, 2000 nM HD22). (b) APTT of human plasma clotting in presence of heparin or low molecular weight heparin (LMWH) with different concentration. (c) PT of human plasma clotting in presence of the mixture of origami and aptamers (Ori + T + H) or Aptarray with different concentration. Data represent the mean ± s.d. from either five (a-b) or three (c) independent replicates.

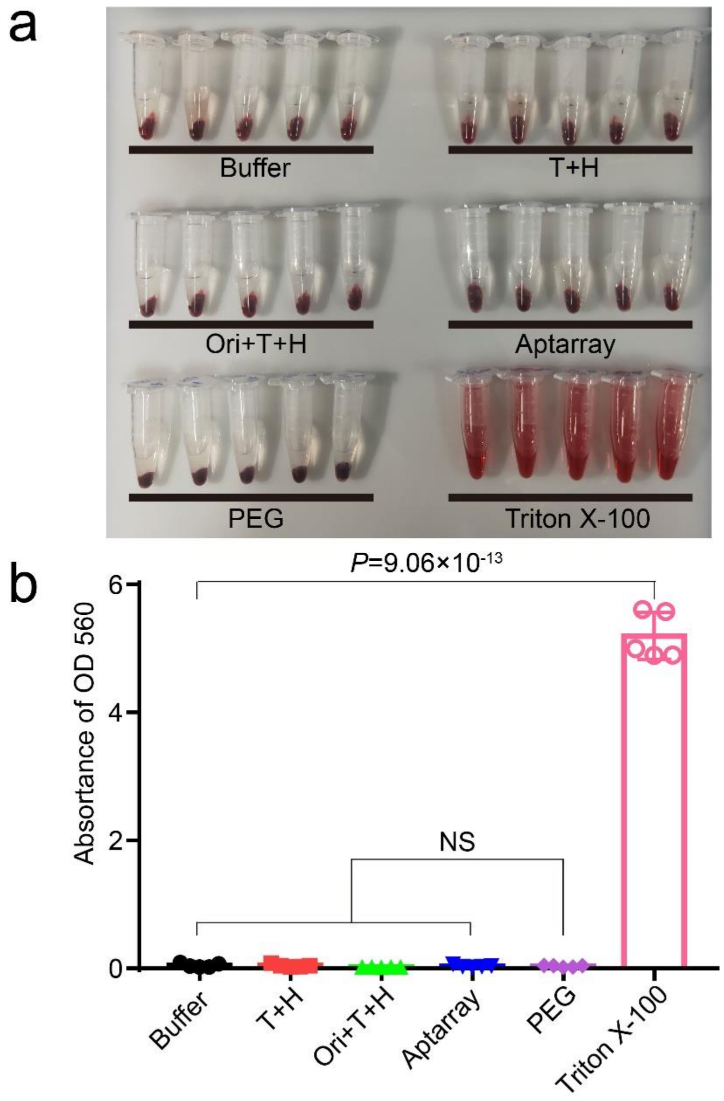

Supplementary Figure 26. Hemolysis assays. (a) The photograph of red blood cells (RBCs) samples treated with buffer, the mixture of aptamers (T + H), the mixture of origami and aptamers (Ori + T + H), Aptarray, the negative control polyethylene glycol 8000 (PEG) and the positive control Triton X-100. (b) The absorbances at 560 nm (A560) of blood samples treated by the indicated groups. Data represent the mean ± s.d. from five independent replicates. NS, $P > 0.05$.

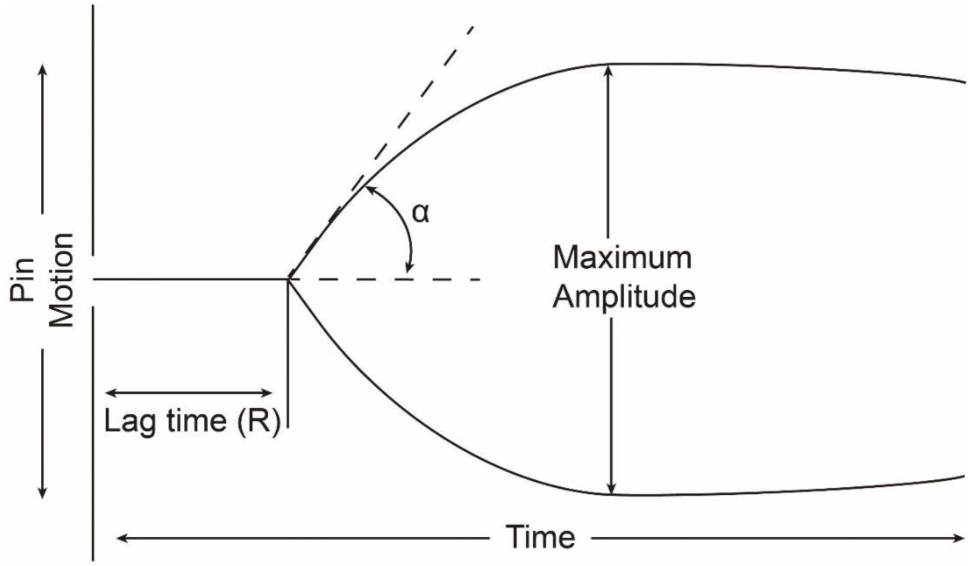

Supplementary Figure 27. The representative (thromboelastography) TEG tracing obtained by TEG machine. Three values that reflect clot formation are determined by TEG assay: the lag time, the alpha-angle and the maximum amplitude (MA). The lag time is the time until the first evidence of a clot is formed. The alpha-angle represents the speed of clot formation. The MA value is a reflection of the mechanical strength of a clot.

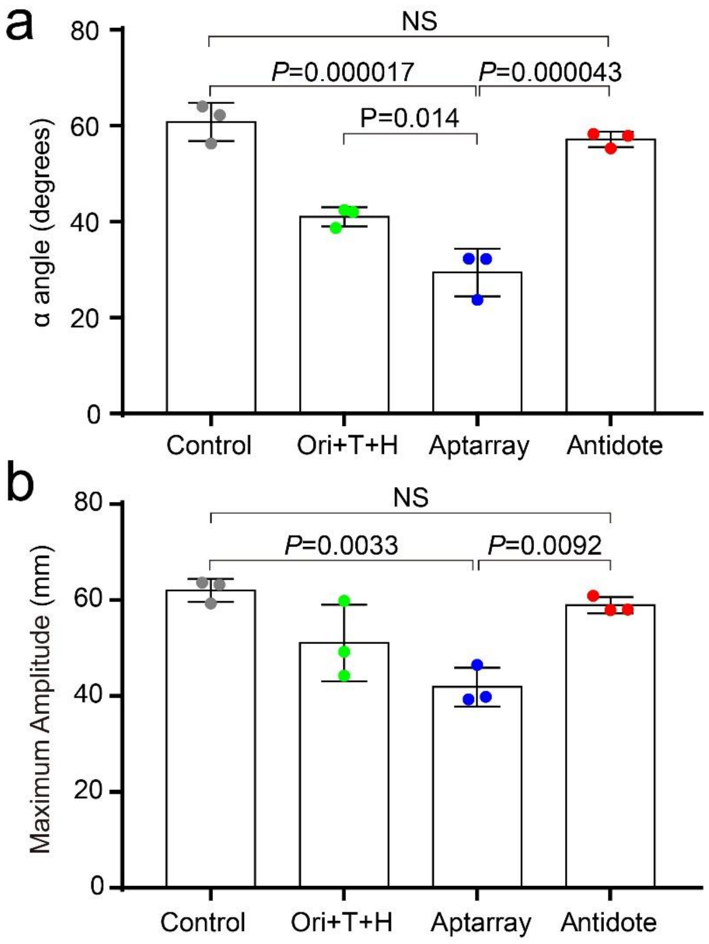

Supplementary Figure 28. The alpha-angle and maximum amplitude (MA) obtained from TEG software. The whole blood samples were in the absence (Control) and presence of the mixture of origami and two types of aptamers (Ori + T + H; origami ~56 nM, TBA15, 2 μM, HD22, 2 μM), Aptarray (origami ~ 56 nM, TBA15, 2 μM, HD22, 2 μM) and 5-fold antidote-neutralized Aptarray (Antidot; cTBA15, 10 μM and cHD22, 10 μM, mixed with Aptarray for 5 min at 25 °C). Data represent the mean ± s.d. from three independent replicates. Statistical significance was calculated by one-way ANOVA with the Tukey post hoc test. NS, $P > 0.05$.

.

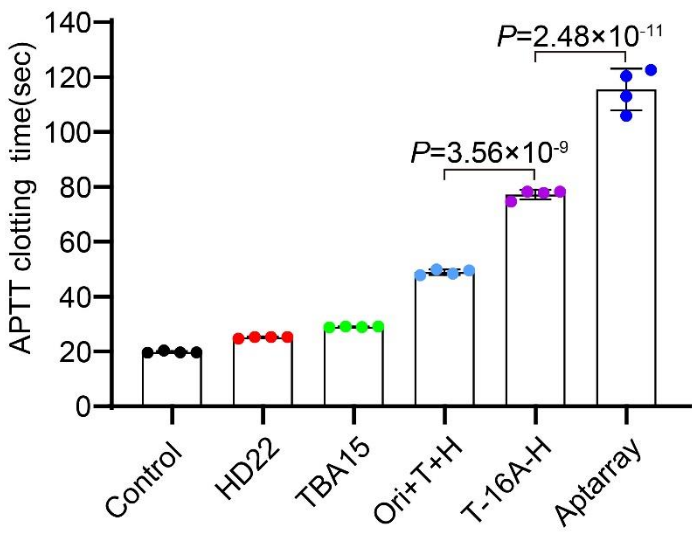

Supplementary Figure S29. APTT of mice plasma clotting measurements. APTT of mice plasma clotting in absence (Control) or presence of HD22 (2 μM), TBA15 (2 μM), the mixture of origami and aptamers (Ori + T + H, TBA15, 2 μM, HD22, 2 μM and origami ~ 56nM), aptamers tethered with 16-mer linker (T-16A-H, 2 μM) or Aptarray (~ 56 nM). Data represent the mean ± s.d. from four independent replicates. Statistical significance was calculated by one-way ANOVA with the Tukey post hoc test.

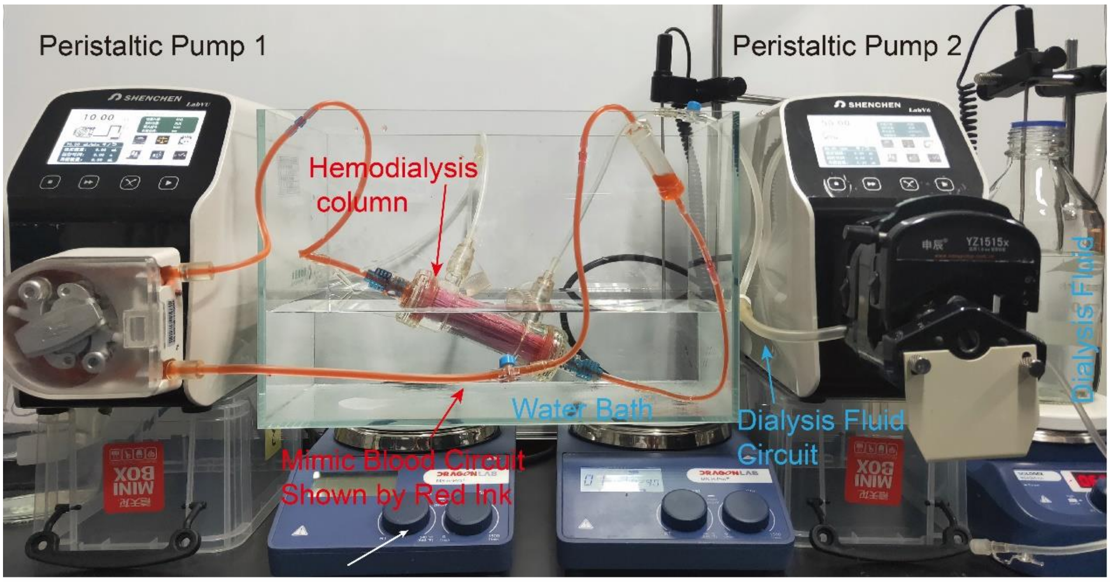

Supplementary Figure 30. A continuous recirculation within an *ex vivo* circuit. Solution (red, mimicking whole blood) was pumped via mechanical roller pump 1 to hemodialysis column through the closed extracorporeal circuit. Colorless and transparent solution (dialysate) was motivated by pump 2 and flowed through the device and then into a waste container. The temperature of the dialyzer and dialysate was maintained at ~ 30°C.

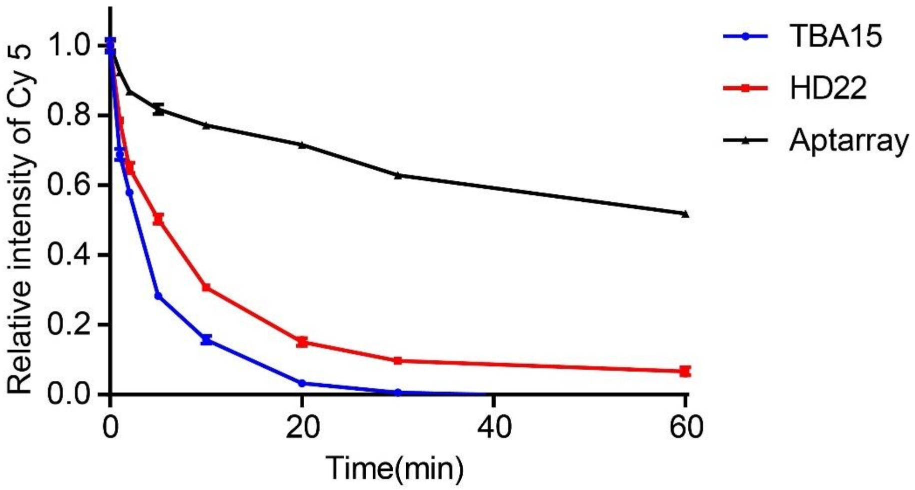

Supplementary Figure 31. Clearance of Cy5-labelled aptamers v.s. dye-containing Aptarray. Data represent the mean ± s.d. from three independent replicates.

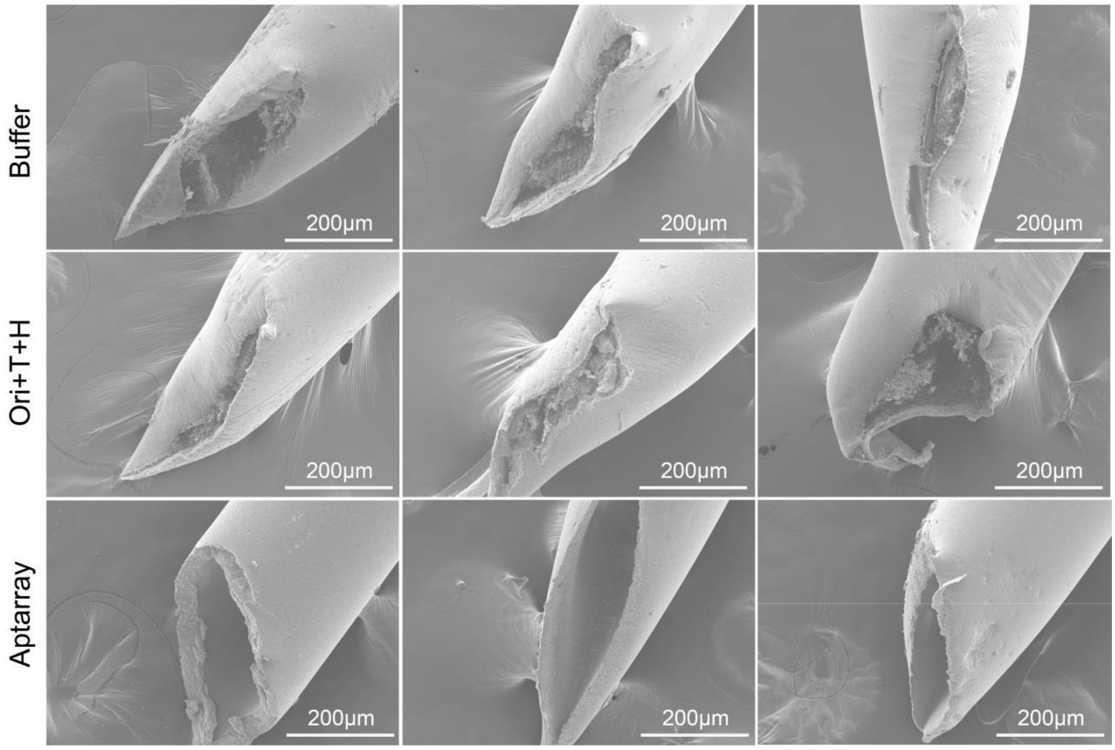

Supplementary Figure 32. Additional SEM images of filters collected from the post circulation dialyzers after different anticoagulation strategies. Scale bars, 200 μm. The SEM images are representative of three independent replicates.

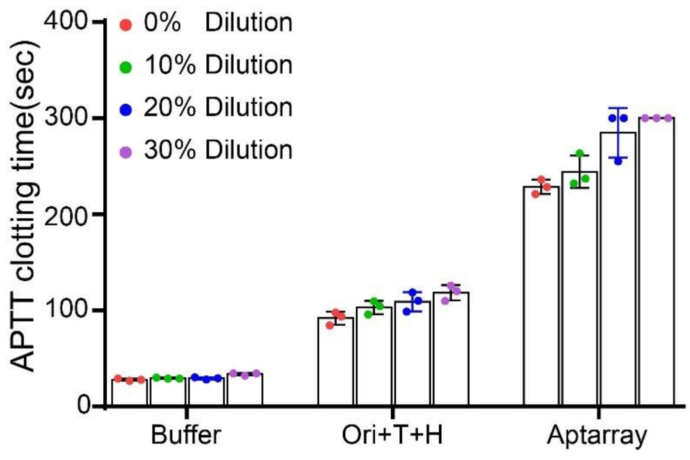

Supplementary Figure 33. The plasma clotting time in the presence of one of the anticoagulant strategies at the different dilution ratios by dialysate solution. Data represent the mean ± s.d. from three independent replicates.

1.4 Safety assessment of the Aptarray

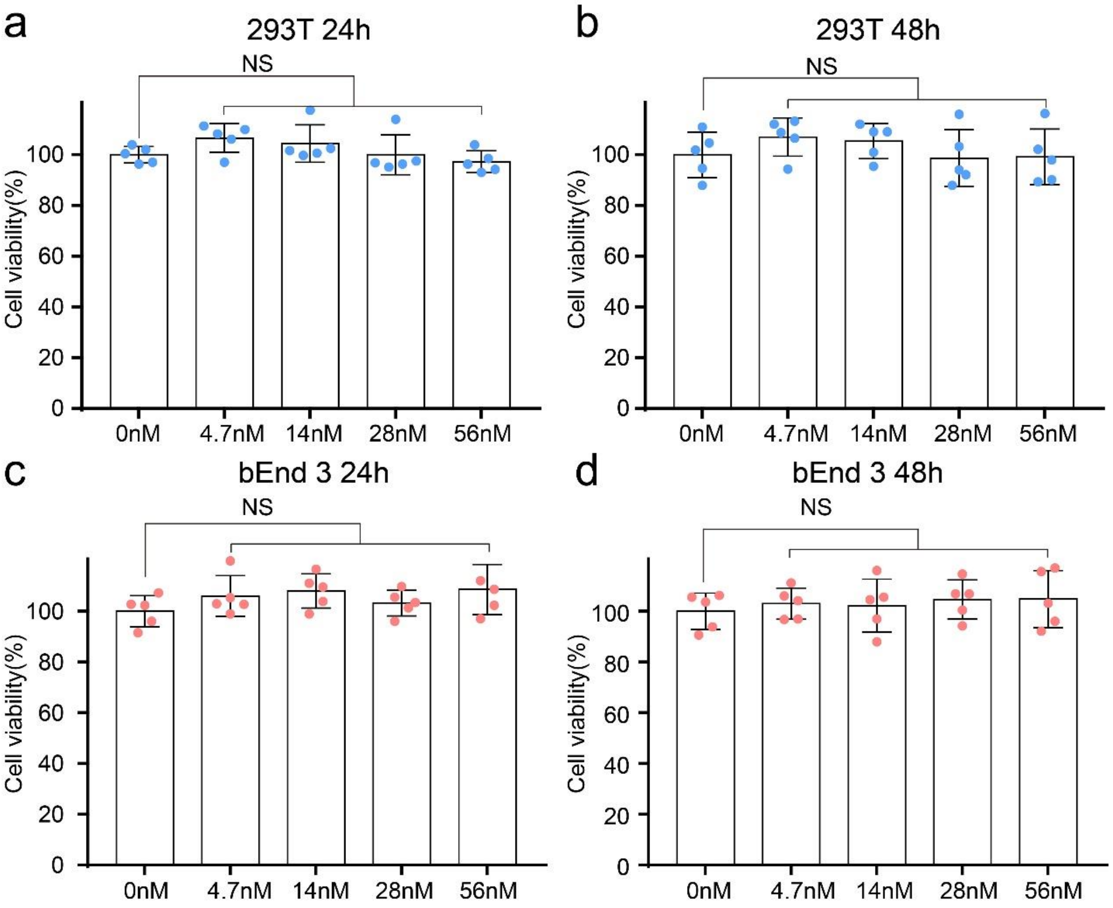

Supplementary Figure 34. Viability of (a-b) HEK 293T and (c-d) b.end3 cells after incubation with Aptarray at indicated concentrations. Data represent the mean ± s.d. from five independent replicates. Statistical significance was calculated by one-way ANOVA with the Tukey post hoc test. NS, $P > 0.05$.

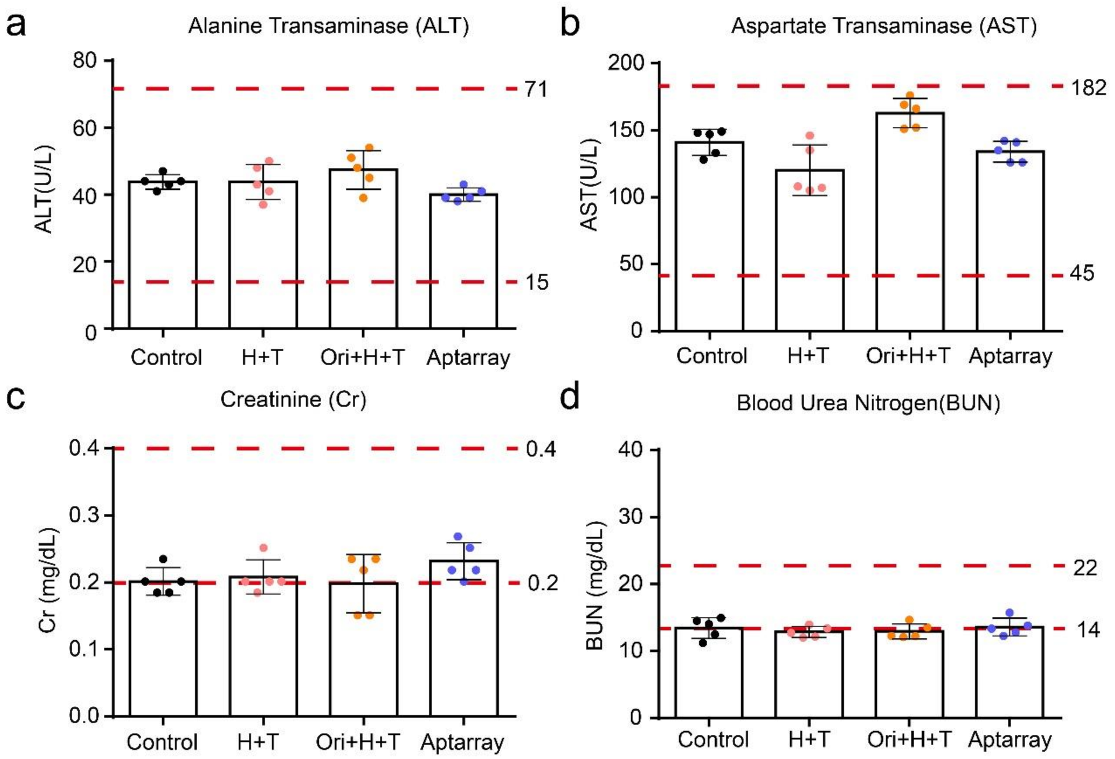

Supplementary Figure 35. Levels of (a) alanine transaminase (ALT), (b) aspartate transaminase (AST), (c) creatinine (Cr) and (d) blood urea nitrogen (BUN) in mice after tail injection with Aptarray (~ 560 nM, 100 μL) or other indicated samples. Data represent the mean ± s.d. from five animals in each group.

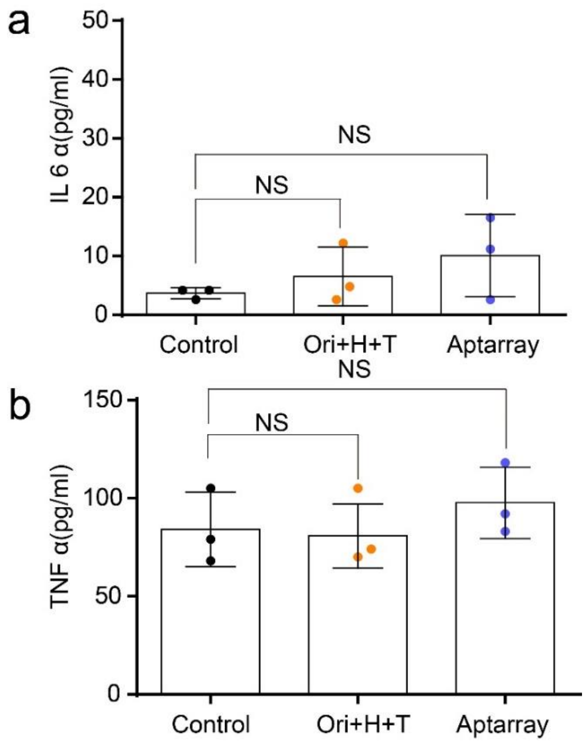

Supplementary Figure 36. Serum cytokine concentrations in mice treated with Aptarray or other indicated controls. Serum levels of (a) interleukin-6 (IL-6) and (b) tumor necrosis factor alpha (TNF-α) were determined. The samples were collected from BABL/c mice treated with buffer (Control), the mixture of origami and aptamers (Ori + T + H, aptamers 20 μM and origami ~ 560 nM) or Aptarray (~ 560 nM) via a single tail vein injection. Data represent the mean ± s.d. from three animals in each group. Statistical significance was calculated by one-way ANOVA with the Tukey post hoc test. NS, $P > 0.05$.

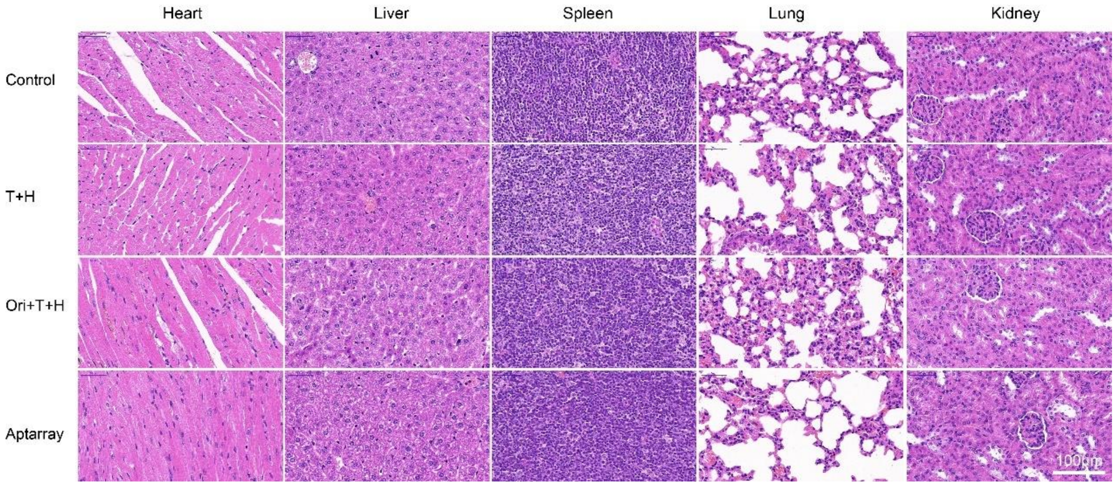

Supplementary Figure 37. Representative H&E staining images of major organs collected from mice injected intravenously with Aptarray or other indicated controls. Scale bar, 100 μm. The images are representative of five independent animals.

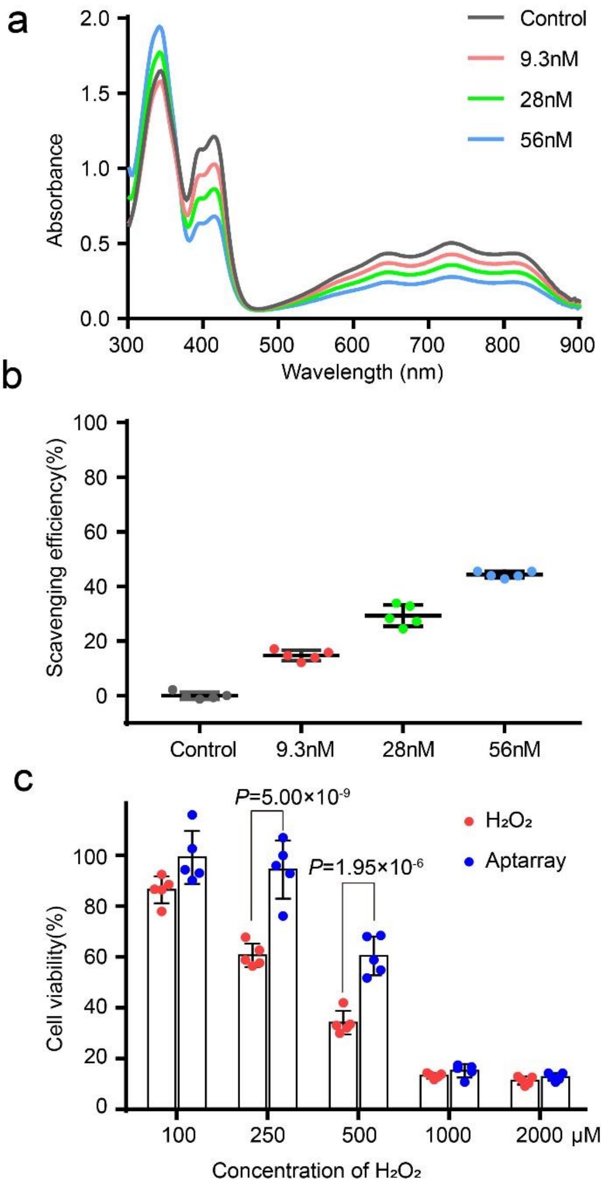

Supplementary Figure 38. ROS scavenging effect of Aptarray in vitro. (a) UV-Vis spectra of ABTS radicals incubated with various concentrations of Aptarray (~ 9.3, 28, 56 nM) for 5 h. (b) Analysis of the ROS scavenging efficiency (%) of Aptarray. The characteristic absorbance of ABTS radicals at 734 nm (A734) decreased after incubation with Aptarray. (c) Cell viability analysis evaluated by MTT assay when incubating HEK293 cells with different concentrations of H_2O_2 (100, 250, 500, 1000, 2000 μM) and Aptarray (56 nM). In (b-c), data represent the mean ± s.d. from five independent replicates. Statistical significance was calculated by one-way ANOVA with the Tukey post hoc test.

Supplementary Table 1. Plasma clotting time in presence of one of the anticoagulant strategies at different indicated time points.

Time	control	Ori+T+H/APTT			Aptarray/APTT		
1 min	Failed to readout	134.5s	133.7s	134.1s	258.4s	265.5s	262.9s
5 min		97.8s	98.7s	96s	263.7s	262.2s	265.1s
10 min		76.4s	78.2s	75.5s	266s	270.8s	271s
20 min		48.6s	49.6s	48.2s	300s	300s	300s

Supplementary Table 2

Sequences of staple strand pool and functional strands

All the sequences are present from 5'-3' (left to right).

Sequence of thrombin binding aptamers and antidote strands	
TBA15	GGTTGGTGTGGTTGG
HD22	TAGTCCGTGGTAGGGCAGGTTGGGGTGACT
TTT-TBA15	TTTTTTTTTTTTTTTACGCGGTTGGTGTGGTTGG
TAT-HD22	TATTATTATTATTATTTTAGTCCGTGGTAGGGCAGGTTGGGGTGACT
cTBA 15 (antidote strand)	CCAACCACACCAACCGCGTAAAAAAAAAAAAAAA
cHD22 (antidote strand)	AGTCACCCCAACCTGCCCTACCACGGACTAAAAATAATAATAATAATA
Sequence of unmodified staple strands of square origami	
S13	TGGTTTTTAACGTCAAAGGGCGAAGAACCATC
S14	CTTGCATGCATTAATGAATCGGCCCGCCAGGG
S15	TAGATGGGGGGTAACGCCAGGGTTGTGCCAAG
S16	CATGTCAAGATTCTCCGTGGGAACCGTTGGTG
S17	CTGTAATATTGCCTGAGAGTCTGGAAAACTAG
S18	TGCAACTAAGCAATAAAGCCTCAGTTATGACC
S19	AAACAGTTGATGGCTTAGAGCTTATTTAAATA
S20	ACGAACTAGCGTCCAATACTGCGGAATGCTTT
S21	CTTTGAAAAGAACTGGCTCATTATTTAATAAA
S22	ACGGCTACTTACTTAGCCGGAACGCTGACCAA
S23	GAGAATAGCTTTTGCGGGATCGTCGGGTAGCA
S24	ACGTTAGTAAATGAATTTTCTGTAAGCGGAGT
S25	ACCCAAATCAAGTTTTTTGGGGTCAAAGAACG
S26	TGGACTCCCTTTTCACCAGTGAGACCTGTCGT
S27	GCCAGCTGCCTGCAGGTCGACTCTGCAAGGCG
S28	ATTAAGTTCGCATCGTAACCGTGCGAGTAACA
S29	ACCCGTCGTCATATGTACCCCGGTAAAGGCTA
S30	TCAGGTCACTTTTGCGGGAGAAGCAGAATTAG
S31	CAAAATTAAAGTACGGTGTCTGGAAGAGGTCA
S32	TTTTTGCGCAGAAAACGAGAATGAATGTTTAG
S33	ACTGGATAACGGAACAACATTATTACCTTATG
S34	CGATTTTAGAGGACAGATGAACGGCGCGACCT
S35	GCTCCATGAGAGGCTTTGAGGACTAGGGAGTT
S36	AAAGGCCGAAAGGAACAACTAAAGCTTTCCAG
S37	AGCTGATTACAAGAGTCCACTATTGAGGTGCC
S38	CCCGGGTACTTTCCAGTCGGGAAACGGGCAAC
S39	GTTTGAGGGAAAGGGGGATGTGCTAGAGGATC
S40	AGAAAAGCAACATTAAATGTGAGCATCTGCCA
S41	CAACGCAATTTTTGAGAGATCTACTGATAATC
S42	TCCATATACATACAGGCAAGGCAACTTTATTT

S43	CAAAAATCATTGCTCCTTTTGATAAGTTTCAT
S44	AAAGATTCAGGGGGTAATAGTAAACCATAAAT
S45	CCAGGCGCTTAATCATTGTGAATTACAGGTAG
S46	TTTCATGAAAATTGTGTCGAAATCTGTACAGA
S47	AATAATAAGGTCGCTGAGGCTTGCAAAGACTT
S48	CGTAACGATCTAAAGTTTTGTCGTGAATTGCG
S49	GTAAAGCACTAAATCGGAACCCTAGTTGTTCC
S50	AGTTTGGAGCCCTTCACCGCCTGGTTGCGCTC
S51	ACTGCCCGCCGAGCTCGAATTCGTTATTACGC
S52	CAGCTGGCGGACGACGACAGTATCGTAGCCAG
S53	CTTTCATCCCCAAAAACAGGAAGACCGGAGAG
S54	GGTAGCTAGGATAAAAATTTTTAGTTAACATC
S55	CAATAAATACAGTTGATTCCCAATTTAGAGAG
S56	TACCTTTAAGGTCTTTACCCTGACAAAGAAGT
S57	TTTGCCAGATCAGTTGAGATTTAGTGGTTTAA
S58	TTTCAACTATAGGCTGGCTGACCTTGTATCAT
S59	CGCCTGATGGAAGTTTCCATTAAACATAACCG
S60	ATATATTCTTTTTTCACGTTGAAAATAGTTAG
S61	GAGTTGCACGAGATAGGGTTGAGTAAGGGAGC
S62	TCATAGCTACTCACATTAATTGCGCCCTGAGA
S63	GAAGATCGGTGCGGGCCTCTTCGCAATCATGG
S64	GCAAATATCGCGTCTGGCCTTCCTGGCCTCAG
S65	TATATTTTAGCTGATAAATTAATGTTGTATAA
S66	CGAGTAGAACTAATAGTAGTAGCAAACCCTCA
S67	TCAGAAGCCTCCAACAGGTCAGGATCTGCGAA
S68	CATTCAACGCGAGAGGCTTTTGCATATTATAG
S69	AGTAATCTTAAATTGGGCTTGAGAGAATACCA
S70	ATACGTAAAAGTACAACGGAGATTTCATCAAG
S71	AAAAAAGGACAACCATCGCCCACGCGGGTAAA
S72	TGTAGCATTCCACAGACAGCCCTCATCTCCAA
S73	CCCCGATTTAGAGCTTGACGGGGAAATCAAAA
S74	GAATAGCCGCAAGCGGTCCACGCTCCTAATGA
S75	GTGAGCTAGTTTCCTGTGTGAAATTTGGGAAG
S76	GGCGATCGCACTCCAGCCAGCTTTGCCATCAA
S77	AAATAATTTTAAATTGTAAACGTTGATATTCA
S78	ACCGTTCTAAATGCAATGCCTGAGAGGTGGCA
S79	TCAATTCTTTTAGTTTGACCATTACCAGACCG
S80	GAAGCAAAAAAGCGGATTGCATCAGATAAAAA
S81	CCAAAATATAATGCAGATACATAAACACCAGA
S82	ACGAGTAGTGACAAGAACCGGATATACCAAGC
S83	GCGAAACATGCCACTACGAAGGCATGCGCCGA
S84	CAATGACACTCCAAAAGGAGCCTTACAACGCC
S85	CCAGCAGGGGCAAAATCCCTTATAAAGCCGGC

S86	GCTCACAATGTAAAGCCTGGGGTGGGTTTGCC
S87	GCTTCTGGTCAGGCTGCGCAACTGTGTTATCC
S88	GTTAAAATTTTAACCAATAGGAACCCGGCACC
S89	AGGTAAAGAAATCACCATCAATATAATATTTT
S90	TCGCAAATGGGGCGCGAGCTGAAATAATGTGT
S91	AAGAGGAACGAGCTTCAAAGCGAAGATACATT
S92	GGAATTACTCGTTTACCAGACGACAAAAGATT
S93	CCAAATCACTTGCCCTGACGAGAACGCCAAAA
S94	AAACGAAATGACCCCCAGCGATTATTCATTAC
S95	TCGGTTTAGCTTGATACCGATAGTCCAACCTA
S96	TGAGTTTCGTCACCAGTACAAACTTAATTGTA
S97	GAACGTGGCGAGAAAGGAAGGGAACAAACTAT
S98	CCGAAATCCGAAAATCCTGTTTGAAGCCGGAA
S99	GCATAAAGTTCCACACAACATACGAAGCGCCA
S100	TTCGCCATTGCCGGAAACCAGGCATTAAATCA
S101	GCTCATTTTCGCATTAAATTTTTGAGCTTAGA
S102	AGACAGTCATTCAAAAGGGTGAGAAGCTATAT
S103	TTTCATTTGGTCAATAACCTGTTTATATCGCG
S104	TTTTAATTGCCCGAAAGACTTCAAAACACTAT
S105	CATAACCCGAGGCATAGTAAGAGCTTTTTAAG
S106	GAATAAGGACGTAACAAAGCTGCTCTAAAACA
S107	CTCATCTTGAGGCAAAAGAATACAGTGAATTT
S108	CTTAAACATCAGCTTGCTTTCGAGCGTAACAC
S109	ACGAACCAAAACATCGCCATTAAATGGTGGTT
S110	CGACAACTAAGTATTAGACTTTACAATACCGA
S111	CTTTTACACAGATGAATATACAGTAAACAATT
S112	TTAAGACGTTGAAAACATAGCGATAACAGTAC
S113	GCGTTATAGAAAAAGCCTGTTTAGAAGGCCGG
S114	ATCGGCTGCGAGCATGTAGAAACCTATCATAT
S115	CCTAATTTACGCTAACGAGCGTCTAATCAATA
S116	AAAAGTAATATCTTACCGAAGCCCTTCCAGAG
S117	TTATTCATAGGGAAGGTAAATATTCATTCAGT
S118	GAGCCGCCCCACCACCGGAACCGCGACGGAAA
S119	AATGCCCCGTAACAGTGCCCGTATCTCCCTCA
S120	CAAGCCCAATAGGAACCCATGTACAAACAGTT
S121	CGGCCTTGCTGGTAATATCCAGAACGAACTGA
S122	TAGCCCTACCAGCAGAAGATAAAAACATTTGA
S123	GGATTTAGCGTATTAAATCCTTTGTTTTCAGG
S124	TTTAACGTTCGGGAGAAACAATAATTTTCCCT
S125	TAGAATCCCTGAGAAGAGTCAATAGGAATCAT
S126	AATTACTACAAATTCTTACCAGTAATCCCATC
S127	CTAATTTATCTTTCCTTATCATTCATCCTGAA
S128	TCTTACCAGCCAGTTACAAAATAAATGAAATA

S129	GCAATAGCGCAGATAGCCGAACAATTCAACCG
S130	ATTGAGGGTAAAGGTGAATTATCAATCACCGG
S131	AACCAGAGACCCTCAGAACCGCCAGGGGTCAG
S132	TGCCTTGACTGCCTATTTCGGAACAGGGATAG
S133	AGGCGGTCATTAGTCTTTAATGCGCAATATTA
S134	TTATTAATGCCGTCAATAGATAATCAGAGGTG
S135	CCTGATTGAAAGAAATTGCGTAGACCCGAACG
S136	ATCAAAATCGTCGCTATTAATTAACGGATTCG
S137	ACGCTCAAAATAAGAATAAACACCGTGAATTT
S138	GGTATTAAGAACAAGAAAAATAATTAAAGCCA
S139	ATTATTTAACCCAGCTACAATTTTCAAGAACG
S140	GAAGGAAAATAAGAGCAAGAAACAACAGCCAT
S141	GACTTGAGAGACAAAAGGGCGACAAGTTACCA
S142	GCCACCACTCTTTTCATAATCAAACCGTCACC
S143	CTGAAACAGGTAATAAGTTTTAACCCCTCAGA
S144	CTCAGAGCCACCACCCTCATTTTCCTATTATT
S145	CCGCCAGCCATTGCAACAGGAAAAATATTTTT
S146	GAATGGCTAGTATTAACACCGCCTCAACTAAT
S147	AGATTAGATTTAAAAGTTTGAGTACACGTAAA
S148	ACAGAAATCTTTGAATACCAAGTTCCTTGCTT
S149	CTGTAAATCATAGGTCTGAGAGACGATAAATA
S150	AGGCGTTACAGTAGGGCTTAATTGACAATAGA
S151	TAAGTCCTACCAAGTACCGCACTCTTAGTTGC
S152	TATTTTGCTCCCAATCCAAATAAGTGAGTTAA
S153	GCCCAATACCGAGGAAACGCAATAGGTTTACC
S154	AGCGCCAACCATTTGGGAATTAGATTATTAGC
S155	GTTTGCCACCTCAGAGCCGCCACCGATACAGG
S156	AGTGTACTTGAAAGTATTAAGAGGCCGCCACC
S157	GCCACGCTATACGTGGCACAGACAACGCTCAT
S158	ATTTTGCGTCTTTAGGAGCACTAAGCAACAGT
S159	GCGCAGAGATATCAAAATTATTTGACATTATC
S160	TAACCTCCATATGTGAGTGAATAAACAAAATC
S161	CATATTTAGAAATACCGACCGTGTTACCTTTT
S162	CAAGCAAGACGCGCCTGTTTATCAAGAATCGC
S163	TTTTGTTTAAGCCTTAAATCAAGAATCGAGAA
S164	ATACCCAAGATAACCCACAAGAATAAACGATT
S165	AATCACCAAATAGAAAATTCATATATAACGGA
S166	CACCAGAGTTCGGTCATAGCCCCCGCCAGCAA
S167	CCTCAAGAATACATGGCTTTTGATAGAACCAC
S168	CCCTCAGAACCGCCACCCTCAGAACTGAGACT
S169	GGAAATACCTACATTTTGACGCTCACCTGAAA
S170	GCGTAAGAGAGAGCCAGCAGCAAAAAGGTTAT
S171	CTAAAATAGAACAAAGAAACCACCAGGGTTAG

S172	AACCTACCGCGAATTATTCATTTCCAGTACAT
S173	AAATCAATGGCTTAGGTTGGGTTACTAAATTT
S174	AATGGTTTACAACGCCAACATGTAGTTCAGCT
S175	AATGCAGACCGTTTTTATTTTCATCTTGCGGG
S176	AGGTTTTGAACGTCAAAAATGAAAGCGCTAAT
S177	ATCAGAGAAAGAACTGGCATGATTTTATTTTG
S178	TCACAATCGTAGCACCATTACCATCGTTTTCA
S179	TCGGCATTCCGCCGCCAGCATTGACGTTCCAG
S180	TAAGCGTCGAAGGATTAGGATTAGTACCGCCA
S181	CTAAAGCAAGATAGAACCCTTCTGAATCGTCT
S182	CGGAATTATTGAAAGGAATTGAGGTGAAAAAT
S183	GAGCAAAAACTTCTGAATAATGGAAGAAGGAG
S184	TATGTAAACCTTTTTTAATGGAAAAATTACCT
S185	AGAGGCATAATTTCATCTTCTGACTATAACTA
S186	TCATTACCCGACAATAAACAACATATTTAGGC
S187	CTTTACAGTTAGCGAACCTCCCGACGTAGGAA
S188	TTATTACGGTCAGAGGGTAATTGAATAGCAGC
S189	CCGGAAACACACCACGGAATAAGTAAGACTCC
S190	TGAGGCAGGCGTCAGACTGTAGCGTAGCAAGG
S191	TGCTCAGTCAGTCTCTGAATTTACCAGGAGGT
S192	TATCACCGTACTCAGGAGGTTTAGCGGGGTTT
S193	GAAATGGATTATTTACATTGGCAGACATTCTG
S194	GCCAACAGTCACCTTGCTGAACCTGTTGGCAA
S195	ATCAACAGTCATCATATTCCTGATTGATTGTT
S196	TGGATTATGAAGATGATGAAACAAAATTTCAT
S197	TTGAATTATGCTGATGCAAATCCACAAATATA
S198	TTTTAGTTTTTCGAGCCAGTAATAAATTCTGT
S199	CCAGACGAGCGCCCAATAGCAAGCAAGAACGC
S200	GAGGCGTTAGAGAATAACATAAAAGAACACCC
S201	TGAACAAACAGTATGTTAGCAAACTAAAAGAA
S202	ACGCAAAGGTCACCAATGAAACCAATCAAGTT
S203	TGCCTTTAGTCAGACGATTGGCCTGCCAGAAT
S204	GGAAAGCGACCAGGCGGATAAGTGAATAGGTG
Staple strands along the two wide sides are extended with TTTTT at their 5’-ends	
5t1	TTTTTCGATGGCCCACTACGTAAACCGTC
5t2	TTTTTCGGTTTGCGTATTGGGAACGCGCG
5t5	TTTTTGATGAACGGTAATCGTAGCAAACA
5t6	TTTTTGGTTGTACCAAAAACAAGCATAAA
5t7	TTTTTCTGTAGCTCAACATGTATTGCTGA
5t8	TTTTTCATTGAATCCCCCTCAAATCGTCA
5t11	TTTTTGACAGCATCGGAACGAACCCTCAG
5t12	TTTTTACTTTCAACAGTTTCTGGGATTTT
5t205	TTTTTACCAGTAATAAAAGGGATTCACCA

5t206	TTTTTAATCAATATCTGGTCACAAATATC
5t209	TTTTTCGCGAGAAAACTTTTTATCGCAAG
5t210	TTTTTCGACAAAAGGTAAAGTAGAGAATA
5t211	TTTTTGCTTATCCGGTATTCTAAATCAGA
5t212	TTTTTGACGGGAGAATTAACTACAGGGAA
5t215	TTTTTATAAATCCTCATTAAATGATATTC
5t216	TTTTTTATAAGTATAGCCCGGCCGTCGAG
Capture strands for aptamer TBA15 loading:	
AAA27	AAAAAAAAAAAAAAAAGCCAGCTGCCTGCAGGTCGACTCTGAAGGCG
AAA28	AAAAAAAAAAAAAAAAATTAAGTTCGCATCGTAACCGTGCGAGTAACA
AAA29	AAAAAAAAAAAAAAAAACCCGTCGTCATATGTACCCCGGTAAAGGCTA
AAA30	AAAAAAAAAAAAAAAATCAGGTCACTTTTGCGGGAGAAGCAGAATTAG
AAA31	AAAAAAAAAAAAAAAACAAAATTAAAGTACGGTGTCTGGAAGAGGTCA
AAA32	AAAAAAAAAAAAAAAATTTTTGCGCAGAAAACGAGAATGAATGTTTAG
AAA33	AAAAAAAAAAAAAAAAACTGGATAACGGAACAACATTATTACCTTATG
AAA34	AAAAAAAAAAAAAAAACGATTTTAGAGGACAGATGAACGGCGCGACCT
AAA35	AAAAAAAAAAAAAAAAGCTCCATGAGAGGCTTTGAGGACTAGGGAGTT
AAA75	AAAAAAAAAAAAAAAAGTGAGCTAGTTTCCTGTGTGAAATTTGGGAAG
AAA76	AAAAAAAAAAAAAAAAGGCGATCGCACTCCAGCCAGCTTTGCCATCAA
AAA77	AAAAAAAAAAAAAAAAAAATAATTTTAAATTGTAAACGTTGATATTCA
AAA78	AAAAAAAAAAAAAAAAACCGTTCTAAATGCAATGCCTGAGAGGTGGCA
AAA79	AAAAAAAAAAAAAAAATCAATTCTTTTAGTTTGACCATTACCAGACCG
AAA80	AAAAAAAAAAAAAAAAGAAGCAAAAAAGCGGATTGCATCAGATAAAAA
AAA81	AAAAAAAAAAAAAAAACCAAAATATAATGCAGATACATAAACACCAGA
AAA82	AAAAAAAAAAAAAAAAACGAGTAGTGACAAGAACCGGATATACCAAGC
AAA83	AAAAAAAAAAAAAAAAGCGAAACATGCCACTACGAA

	GGCATGCGCCGA
AAA123	AAAAAAAAAAAAAAAAGGATTTAGCGTATTAAATCCT TTGTTTTCAGG
AAA124	AAAAAAAAAAAAAAAATTTAACGTTCGGGAGAAACA ATAATTTTCCCT
AAA125	AAAAAAAAAAAAAAAATAGAATCCCTGAGAAGAGTC AATAGGAATCAT
AAA126	AAAAAAAAAAAAAAAAAATTACTACAAATTCTTACCA GTAATCCCATC
AAA127	AAAAAAAAAAAAAAAACTAATTTATCTTTCCTTATCAT TCATCCTGAA
AAA128	AAAAAAAAAAAAAAAATCTTACCAGCCAGTTACAAAA TAAATGAAATA
AAA129	AAAAAAAAAAAAAAAAGCAATAGCGCAGATAGCCGA ACAATTCAACCG
AAA130	AAAAAAAAAAAAAAAAATTGAGGGTAAAGGTGAATTA TCAATCACCGG
AAA131	AAAAAAAAAAAAAAAAAACCAGAGACCCTCAGAACC GCCAGGGGTCAG
AAA171	AAAAAAAAAAAAAAAACTAAAATAGAACAAAGAAAC CACCAGGGTTAG
AAA172	AAAAAAAAAAAAAAAAAACCTACCGCGAATTATTCAT TTCCAGTACAT
AAA173	AAAAAAAAAAAAAAAAAAATCAATGGCTTAGGTTGGG TTACTAAATTT
AAA174	AAAAAAAAAAAAAAAAAATGGTTTACAACGCCAACAT GTAGTTCAGCT
AAA175	AAAAAAAAAAAAAAAAAATGCAGACCGTTTTTATTTT CATCTTGCGGG
AAA176	AAAAAAAAAAAAAAAAGGTTTTGAACGTCAAAAAT GAAAGCGCTAAT
AAA177	AAAAAAAAAAAAAAAATCAGAGAAAGAACTGGCAT GATTTTATTTTG
AAA178	AAAAAAAAAAAAAAAATCACAATCGTAGCACCATTAC CATCGTTTTCA
AAA179	AAAAAAAAAAAAAAAATCGGCATTCCGCCGCCAGCAT TGACGTTCCAG
Capture strands for aptamer HD22 loading:	
ATAT38	ATAATAATAATAATACCCGGGTACTTTCCAGTCGGGAA ACGGGCAAC
ATAT39	ATAATAATAATAATAGTTTGAGGGAAAGGGGGATGTG CTAGAGGATC
ATAT40	ATAATAATAATAATAAGAAAAGCAACATTAAATGTGA

	GCATCTGCCA
ATAT41	ATAATAATAATAATACAACGCAATTTTTGAGAGATCTACTGATAATC
ATAT42	ATAATAATAATAATATCCATATACATACAGGCAAGGCAACTTTATTT
ATAT43	ATAATAATAATAATACAAAAATCATTGCTCCTTTTGATAAGTTTCAT
ATAT44	ATAATAATAATAATAAAAGATTCAGGGGGTAATAGTAAACCATAAAT
ATAT45	ATAATAATAATAATACCAGGCGCTTAATCATTGTGAATTACAGGTAG
ATAT46	ATAATAATAATAATATTTCATGAAAATTGTGTCGAAATCTGTACAGA
ATAT86	ATAATAATAATAATAGCTCACAATGTAAAGCCTGGGGTGGGTTTGCC
ATAT87	ATAATAATAATAATAGCTTCTGGTCAGGCTGCGCAACTGTGTTATCC
ATAT88	ATAATAATAATAATAGTTAAAATTTTAACCAATAGGAACCCGGCACC
ATAT89	ATAATAATAATAATAAGGTAAAGAAATCACCATCAATATAATATTTT
ATAT90	ATAATAATAATAATATCGCAAATGGGGCGCGAGCTGAAATAATGTGT
ATAT91	ATAATAATAATAATAAAGAGGAACGAGCTTCAAAGCGAAGATACATT
ATAT92	ATAATAATAATAATAGGAATTACTCGTTTACCAGACGACAAAAGATT
ATAT93	ATAATAATAATAATACCAAATCACTTGCCCTGACGAGAACGCCAAAA
ATAT94	ATAATAATAATAATAAAACGAAATGACCCCCAGCGATTATTCATTAC
ATAT134	ATAATAATAATAATATTATTAATGCCGTCAATAGATAATCAGAGGTG
ATAT135	ATAATAATAATAATACCTGATTGAAAGAAATTGCGTAGACCCGAACG
ATAT136	ATAATAATAATAATAATCAAAATCGTCGCTATTAATTAACGGATTCG
ATAT137	ATAATAATAATAATAACGCTCAAAATAAGAATAAACACCGTGAATTT
ATAT138	ATAATAATAATAATAGGTATTAAGAACAAGAAAAATAATTAAAGCCA
ATAT139	ATAATAATAATAATAATTATTTAACCCAGCTACAATTTTCAAGAACG

ATAT140	ATAATAATAATAATAGAAGGAAAATAAGAGCAAGAAACAACAGCCAT
ATAT141	ATAATAATAATAATAGACTTGAGAGACAAAAGGGCGACAAGTTACCA
ATAT142	ATAATAATAATAATAGCCACCACTCTTTTCATAATCAAACCGTCACC
ATAT182	ATAATAATAATAATACGGAATTATTGAAAGGAATTGAGGTGAAAAAT
ATAT183	ATAATAATAATAATAGAGCAAAAACTTCTGAATAATGGAAGAAGGAG
ATAT184	ATAATAATAATAATATATGTAAACCTTTTTTAATGGAAAAATTACCT
ATAT185	ATAATAATAATAATAAGAGGCATAATTTCATCTTCTGACTATAACTA
ATAT186	ATAATAATAATAATATCATTACCCGACAATAAACAACATATTTAGGC
ATAT187	ATAATAATAATAATACTTTACAGTTAGCGAACCTCCCGACGTAGGAA
ATAT188	ATAATAATAATAATATTATTACGGTCAGAGGGTAATTGAATAGCAGC
ATAT189	ATAATAATAATAATACCGGAAACACACCACGGAATAAGTAAGACTCC
ATAT190	ATAATAATAATAATATGAGGCAGGCGTCAGACTGTAGCGTAGCAAGG
