# Supplementary material for: A DNA origami-based aptamer nanoarray for potent and reversible anticoagulation in hemodialysis
Source: Nat Commun. 2021 Jan 13;12:358. doi: 10.1038/s41467-020-20638-7 (PMC7807036; doi:10.1038/s41467-020-20638-7)
Supplement: Supplementary file 3 — Reporting Summary [file 41467_2020_20638_MOESM3_ESM.pdf]

nature research

Corresponding author(s): Baoquan Ding

Last updated by author(s): Dec 4, 2020

Reporting Summary

Nature Research wishes to improve the reproducibility of the work that we publish. This form provides structure for consistency and transparency in reporting. For further information on Nature Research policies, see Authors & Referees and the Editorial Policy Checklist .

Statistics

For all statistical analyses, confirm that the following items are present in the figure legend, table legend, main text, or Methods section.

n/a	Confirmed	
☐	☒	The exact sample size (*n*) for each experimental group/condition, given as a discrete number and unit of measurement
☐	☒	A statement on whether measurements were taken from distinct samples or whether the same sample was measured repeatedly
☐	☒	The statistical test(s) used AND whether they are one- or two-sided<br>*Only common tests should be described solely by name; describe more complex techniques in the Methods section.*
☒	☐	A description of all covariates tested
☒	☐	A description of any assumptions or corrections, such as tests of normality and adjustment for multiple comparisons
☐	☒	A full description of the statistical parameters including central tendency (e.g. means) or other basic estimates (e.g. regression coefficient) AND variation (e.g. standard deviation) or associated estimates of uncertainty (e.g. confidence intervals)
☐	☒	For null hypothesis testing, the test statistic (e.g. *F*, *t*, *r*) with confidence intervals, effect sizes, degrees of freedom and *P* value noted<br>*Give P values as exact values whenever suitable.*
☒	☐	For Bayesian analysis, information on the choice of priors and Markov chain Monte Carlo settings
☒	☐	For hierarchical and complex designs, identification of the appropriate level for tests and full reporting of outcomes
☒	☐	Estimates of effect sizes (e.g. Cohen's *d*, Pearson's *r*), indicating how they were calculated

Our web collection on statistics for biologists contains articles on many of the points above.

Software and code

Policy information about availability of computer code

Data collection	AFM was performed in scan-in-fluid mode (Mutimode-8, Bruker) and was processed using Bruker NanoScope Analysis 1.9 software. Ultraviolet-visible absorption spectra were recorded using a UV-VIS spectrophotometer (UV-2450, Shimadzu) and UVProbe 2.61 software. Scattered light intensity measurements were performed with a fluorescence spectrometer (Cary Eclipse, Agilent Technologies) and OriginPro 9.1 software was used to collect data. Activated partial thromboplastin time (APTT) assays and prothrombin time (PT) were performed using a Sysmex CS-5100 System analyzer (Siemens, Germany) according to the manufacturer's instructions. Clot formation was measured with a Thromboelastograph Analyzer (Haemonetics). The clotting reaction of mice plasma was monitored by a semi-automatic coagulation analyzer (SC 40, STEELLEX). SEM imaging was performed using scanning electron microscope (SEM, Hitachi SU8200, Japan) and the data were collected using Hitachi SU8200 ver.1.18.
Data analysis	AFM images were processed on Bruker NanoScope Analysis software (version number: 1.90). Hydrodynamic measurements were processed by dispersion technology software (Zetasizer Nano ZS, 7.11, Malvern). Scattered light intensity results were analyzed by OriginPro 9.1 software. The lag time, α angle and the maximum amplitude were automatically calculated by TEG analytical software 4.2.3 (Haemonetics). Statistical significance determined with GraphPad Prism 8 software and SPSS 24.

For manuscripts utilizing custom algorithms or software that are central to the research but not yet described in published literature, software must be made available to editors/reviewers. We strongly encourage code deposition in a community repository (e.g. GitHub). See the Nature Research guidelines for submitting code & software for further information.

Data

Policy information about availability of data

All manuscripts must include a data availability statement. This statement should provide the following information, where applicable:

- Accession codes, unique identifiers, or web links for publicly available datasets
- A list of figures that have associated raw data
- A description of any restrictions on data availability

The data that support the findings of this study are available within the paper and its supplementary information files. Additional data and files are available from

the corresponding author upon reasonable request. Source data are provided with this paper.

Field-specific reporting

Please select the one below that is the best fit for your research. If you are not sure, read the appropriate sections before making your selection.

- [x] Life sciences
- [ ] Behavioural & social sciences
- [ ] Ecological, evolutionary & environmental sciences

For a reference copy of the document with all sections, see nature.com/documents/nr-reporting-summary-flat.pdf

Life sciences study design

All studies must disclose on these points even when the disclosure is negative.

Sample size	All biochemical and biological experiments were performed in three replicates or more. Sample sizes were determined on the basis of estimates from preliminary experiments and previous similar work (Adv. Mater. 2019, 1808262; Nat. Biotechnol. 2018, 36, 606-613; Nat. Biotechnol. 2004, 22, 1423-1428), so that reasonable statistical analyses could be conducted.
Data exclusions	No animals and/or data were excluded from the analyses.
Replication	The electrophoresis results, AFM images, SEM images and sample graphs are representative of three independent replicates. For the in vitro experiments, at least three biologically independent experiments were performed unless stated otherwise. All attempts at replication were successful. For the in vivo experiments, at least three animals were used in the statistical analysis for APTT assay, tail transection and bleeding studies as well as safety assessment.
Randomization	In the animal studies, mice were randomly divided into the experimental groups. No other randomizations were performed.<br>Human plasma samples were randomly pulled out from Peking University Fourth School of Clinical Medicine and healthy volunteers were randomly selected for normal blood sample donation.
Blinding	All experimental procedures and quantification of results were done by two independent researchers. All researchers were blinded to group allocation.

Reporting for specific materials, systems and methods

We require information from authors about some types of materials, experimental systems and methods used in many studies. Here, indicate whether each material, system or method listed is relevant to your study. If you are not sure if a list item applies to your research, read the appropriate section before selecting a response.

Materials & experimental systems

n/a	Involved in the study	
x		Antibodies
	x	Eukaryotic cell lines
x		Palaeontology
	x	Animals and other organisms
	x	Human research participants
x		Clinical data

Methods

n/a	Involved in the study	
x		ChIP-seq
x		Flow cytometry
x		MRI-based neuroimaging

Eukaryotic cell lines

Policy information about cell lines

Cell line source(s)	Human embryonic kidney 293T cells (HEK293T) and mouse brain endothelial cells (bEnd.3) were purchased from the American Type Culture Collection (Manassas, VA, USA).
Authentication	Cell lines authentication was performed by short tandem repeat DNA profiling and comparison with reference database.
Mycoplasma contamination	All cells lines were negative for mycoplasma.
Commonly misidentified lines (See ICLAC register)	No commonly misidentified cell lines were used.

Animals and other organisms

Policy information about studies involving animals; ARRIVE guidelines recommended for reporting animal research

Laboratory animals	Female BABL/c mice, 6-8 weeks old
Wild animals	No wild animals were used in these studies.
Field-collected samples	The study did not involve samples collected from the field.
Ethics oversight	Animal protocols related to this study were reviewed and approved by the Institutional Animal Care and Use Committee of National Center for Nanoscience and Technology.

Note that full information on the approval of the study protocol must also be provided in the manuscript.

Human research participants

Policy information about studies involving human research participants

Population characteristics	Human plasma samples were obtained from Peking University Fourth School of Clinical Medicine under the protocol (201904-06) approved by Beijing Jishuitan Hospital Institutional Review Board. Samples were collected from 20–60 years old healthy males or females.<br>Blood was obtained from healthy volunteers under IRB-approved protocols. Human research participation consisted only of the recruitment of healthy, consenting volunteer adults for normal blood sample donation. The volunteers are healthy males from 24 to 32 years old.
Recruitment	All volunteers were recruited according to IRB-approved protocols after obtaining informed consent. Healthy volunteers are willing to undergo optional blood draws for research purposes. Written informed consent was obtained from all participants. No other self-selection criteria existed.
Ethics oversight	Beijing Jishuitan Hospital Institutional Review Board (201904-06); Institutional Ethics Committee, National Center for Nanoscience and Technology of China (NCNSTIEC0068-0109)

Note that full information on the approval of the study protocol must also be provided in the manuscript.
